# Unintended health consequences of Swedish parental leave policy (ParLeHealth): protocol for a quasi-experimental study

**DOI:** 10.1136/bmjopen-2021-049682

**Published:** 2021-06-09

**Authors:** Sol Pia Juárez, Helena Honkaniemi, Amy F Heshmati, Enrico Debiasi, Andrea Dunlavy, Anders Hjern, Mikael Rostila, Eleonora Mussino, Srinivasa Vittal Katikireddi, Ann-Zofie Duvander

**Affiliations:** 1 Department of Public Health Sciences, Stockholm University, Stockholm, Sweden; 2 Centre for Health Equity Studies (CHESS), Stockholm University/Karolinska Institutet, Stockholm, Sweden; 3 Department of Global Public Health, Karolinska Institutet, Stockholm, Sweden; 4 Department of Medicine, Karolinska Institutet, Stockholm, Sweden; 5 Stockholm University Demography Unit (SUDA), Stockholm University, Stockholm, Sweden; 6 MRC/CSO Social & Public Health Sciences Unit, University of Glasgow, Glasgow, UK; 7 Department of Sociology, Mid University, Östersund, Sweden

**Keywords:** mental health, child protection, public health, reproductive medicine

## Abstract

**Introduction:**

Sweden has long been praised for a generous parental leave policy oriented towards facilitating a gender-equitable approach to work and parenting. Yet certain aspects of Swedish parental leave could also be responsible for the maintenance of (or even the increase in) health inequalities. Using a ‘Health in All Policies’ lens, this research project aims to assess the unintended health consequences of various components of Sweden’s parental leave policy, including eligibility for and uptake of earnings based benefits.

**Methods and analysis:**

We will use individual-level data from multiple Swedish registers. Sociodemographic information, including parental leave use, will be retrieved from the total population register, Longitudinal Integration Database for Health Insurance and Labour Market Studies and Social Insurance Agency registers. Health information for parents and children will be retrieved from the patient, prescribed drug, cause of death, medical birth and children’s health registers. We will evaluate parents’ mental, mothers’ reproductive and children’s general health outcomes in relation to several policy reforms aiming to protect parental leave benefits in short birth spacing (the speed premium) and to promote father’s uptake (the father’s quota) and sharing of parental leave days (the double days reform). We will also examine effects of increases in basic parental leave benefit levels. Using quasi-experimental designs, we will compare health outcomes across these reforms and eligibility thresholds with interrupted time series, difference-in-difference and regression discontinuity approaches to reduce the risk of health selection and assess causality in the link between parental leave use and health.

**Ethics and dissemination:**

This project has been granted all necessary ethical permissions from the Stockholm Regional Ethical Review Board (Dnr 2019-04913) for accessing and analysing deidentified data. The final outputs will primarily be disseminated as scientific articles published in open-access, high-impact peer-reviewed international journals, as well as press releases and policy briefs.

**Strengths and limitations of this study**

- This will be the first project to evaluate multiple aspects of Swedish parental leave policy by adopting a health equity perspective and Health in All Policies (HiAP) approach.
- The study will use quasi-experimental methods on total population register data to assess causal links between parental leave uptake and health.
- The study will expand on existing evidence of health effects by considering outcomes not only for mothers and children but also for fathers.
- Analyses will be restricted to studying health outcomes that are available within administrative datasets, thereby precluding assessment of potentially more sensitive self-reported assessments of health.

## Introduction

Childbearing is a life event with significant social and health implications for parents, children and society alike. Among women, childbearing has been considered to be responsible for lower labour market participation and gendered wage gaps (ie, the ‘motherhood penalty’),^1^ resulting in lower-income protection in old age.^1–3^ Childbearing is also a risk factor for various physical and mental health conditions among mothers.^4^ By offering secure time off work for childcare and recovery, parental leave has the potential to reduce social and health inequalities in society. However, given that parental leave eligibility and uptake may vary across social strata, it may also have the potential to (re)produce social inequalities in health.

### The Swedish parental leave system

We report intervention details according to the Template for Intervention Description and Replication for Population Health and Policy interventions(TIDieR-PHP) reporting guidelines.^5^

#### Length and flexibility of parental leave

Sweden has developed into one of the most generous and flexible paid parental leave systems worldwide.^6^ Starting in 1974, parents were entitled to 6 months of leave to be used within 270 days of birth and shared by the parents as they desired. An additional 9 months of leave were introduced incrementally from 1975 to 1989,^6 7^ with an extension to use a portion of the leave until the child turned 8 years old in 1978. In 1995, 1 month of the leave was reserved for each parent, a quota which was further extended by 1 month each in 2002 and 2016.^8 9^ Since 2014, couples have been entitled to use 480 parental leave days up until the child turns 12 years old. Of these days, no more than 96 can be used after the child’s fourth birthday.^7^

We will focus on mothers’ and fathers’ paid parental leave (ie, job-protected leave paid at set benefit levels) rather than unpaid parental leave (ie, job-protected unpaid leave in the first 18 months after birth). Furthermore, we do not examine maternity leave (ie, 2 weeks of mandatory job-protected leave for the mother prior to or after childbirth, which can be unpaid or paid as standard parental leave days), temporary leave, including at birth/adoption (formerly paternity leave; ie, up to 10 days of separate paid, job-protected leave for partners to attend the delivery or assist the mother with infant care) or leave for the care of sick children.^6 7^

#### Parental leave eligibility and compensation

In Sweden, all legally residing parents are eligible to receive parental leave benefits (ie, job-protected leave), including biological parents as well as individuals with child custody.^10^ Individuals must apply for these benefits through the Social Insurance Agency, the governmental authority that decides on and administers insurance and contributions to families and children in Sweden.^11^ The benefits are split into general flat-rate or earnings based benefits, collectively referred to as ‘parental benefits’ and applied to 390 out of the total 480 days, while the remaining 90 days are paid at a second flat rate, henceforth referred to as ‘guarantee days’.^7^

To be eligible for earnings based parental benefits, an individual must have been employed for at least 240 consecutive days prior to the estimated delivery date with a minimum annual income of 82 100 Swedish kronor (SEK) (in 2020). Although the remuneration level has varied over time, individuals today receive approximately 80% of their salary up to a certain ceiling (1006 SEK per day in 2020) from the Social Insurance Agency, with potential top-ups from the employer.^12^ If an individual has an income below a certain level (117 590 SEK in 2020) during the 240 days before childbirth, they are only entitled to a general flat-rate benefit (250 SEK per day in 2020) for the first 180 days which they claim for the child. After 180 claimed days, parents can receive benefits based on their current earnings.^7^ Finally, if an individual has no income or an annual income below the stipulated level throughout the leave period, they will receive the general flat-rate benefit for the full 390 days. However, if an individual is sick, a student or unemployed but a ‘job seeker’, they can receive parental leave benefits based on their prior income if they have registered with the Public Employment Service.^10^ Regardless of employment history, the final 90 days are always paid at a minimum guaranteed level of 60 SEK starting in 1987 and 180 SEK since 2006.^7^

#### Policy reforms

The Swedish parental leave system has undergone several reforms over the past half-century. We define a ‘policy reform’ as any change implemented at a specific time that could impact either uptake or sharing of parental leave, as well as eligibility for earnings based benefits. To evaluate the unforeseen health consequences of the parental leave policy—that is, a welfare policy that does not primarily target health—we will concentrate on reforms that have either been shown to accomplish their primary aim (ie, the speed premium, father’s quota and double days reforms) or with undoubtedly significant, although unintended, impacts (ie, the doubled value of general flat-rate benefits).

Doubled flat-rate benefits: Although not explicitly a reform, the doubling of the general flat-rate benefits from 60 SEK per day (from 1987) to 120 SEK per day (in 2002) aimed to secure the economic welfare of low-income families.

Speed premium: Since earnings related parental leave benefits are estimated based on one’s salary prior to childbearing (at approximately 80% of the salary), parents having children in quick succession may choose to temporarily reduce their working hours or be unable to return to work at all and will thus have lower benefits for their second and subsequent births. In 1980, a supplement (commonly referred to as the ‘speed premium’) was introduced to safeguard women’s income between parental leave periods, ensuring that for births with up to 24 months’ spacing^13^ and then with up to 30 months’ spacing (following a 1986 expansion),^14^ all earnings based benefits could be based on the parents’ salaries prior to the birth of their previous child, if higher than their current income. An evaluation of the 1986 expansion revealed that an increased proportion of Swedish-born parents had children in short succession; even if this was an unintended consequence, the reform has henceforth been called the ‘speed premium’.^14^

Father’s quota: Fathers have been eligible to use parental leave since 1974, when parents could choose to divide parental leave days between themselves as they saw fit. However, most days were transferred to and claimed by mothers, with an average use of 320 days in 1994, reinforcing mothers’ ‘double burden’ of childcare and work. In response, a father’s quota reform was implemented in 1995, reserving a month of leave exclusively for the father, to be forfeited completely if left unused (similarly, a 30-day reserve was allocated to the mother, ie, a mother’s quota). It succeeded in increasing the proportion of fathers using parental leave from 43% to 75%.^15^ The father's quota was expanded to 2 and 3 months in 2002 and 2016, respectively, but with smaller impacts on uptake.^6^

Double days: In 2012, 30 days of existing parental leave were designated as ‘shareable’, that is, could be used simultaneously by both parents within the child’s first year of life. By granting flexibility to the couple in their leave use, this ‘double days’ reform aimed to facilitate the transfer of care from one parent to another. In fact, the reform significantly increased the proportion and number of father’s leave days used in the first half-year after childbirth.^16^

All reforms were implemented nationally on 1 January of the indicated year.

### Unintended health consequences of the Swedish parental leave system

Despite the relative generosity of the Swedish parental leave system and its efforts to promote gender equity, some aspects could be responsible for the maintenance or increase in social and health inequities. For example, the strong work-eligibility requirement for earnings based parental leave benefits may exclude groups with less stability in the labour market, such as students and migrants, from benefits equal to those enjoyed by persons with stable incomes.^17^

Inequities in the work-eligibility requirement can emerge in various ways, either by restricting the parental leave benefits scheme to the securely employed and coincidentally excluding parents with ill health (ie, health selection) or by creating health inequities through the unequal distribution of benefits (ie, social causation). For example, individuals with long-term illness may be more likely to have a weak labour market attachment, which in turn can exclude them from earnings related parental leave benefits, thus further increasing socioeconomic inequities. Based on this, we expect that the doubled flat-rate benefits could increase leave uptake among lower-income families or decrease the economic trade-off of using parental leave among lower-income families, thus having potential psychosocial benefits.

Parental leave policies might impact health differently following certain policy reforms. For example, the speed premium supplement introduced to secure earnings based parental leave benefits to parents who have children in quick succession essentially incentivises short birth spacing.^14^ This might lead to increased psychosocial stress and adverse reproductive outcomes among women who become pregnant while simultaneously rearing an infant or toddler. The WHO recommends a minimum of 24 months, assuming 9 months of gestation, before a new conception to avoid adverse reproductive outcomes.^18^ Short birth intervals have been associated with nutrition depletion,^19^ as the mother might not have enough time between births to recover her nutritional reserves and higher risks of weight gain and obesity throughout life.^20^ In turn, these issues are risk factors for adverse outcomes in subsequent children, including low birth weight, preterm birth and perinatal death.^18 21^

The father’s quota was implemented to promote a gender-equitable work-life balance and shared parenting. However, the health consequences of the father’s quota have been debated, with a lack of empirical evidence on its differing mechanisms. Studies have shown a positive association between the father’s parental leave use and healthy behaviours, including decreased alcohol use and increased physical activity,^22 23^ as well as decreased mortality risks.^24^ Nonetheless, there may be situations in which fathers’ leave use is less optimal for or even unfavourable for the family. The quota may have encouraged mothers to return to work sooner, potentially interrupting their physical and psychological recovery from pregnancy and childbirth. These potential adverse effects among parents could extend to the child: shorter maternity leave in the child’s first months could shorten breast feeding, a risk factor for childhood obesity^25^ and respiratory track infectious in the first 2 years of life.^26^ However, there is a lack of evidence on changes in mothers’ labour supply following the reform,^27 28^ suggesting that mothers may have stayed at home with fathers on an unpaid basis, foregoing their income and thus potentially creating psychological strain, especially among low-income parents.

Given that parental leave in Sweden is longer than in most other contexts, it can be argued that any health effect of shortened parental leave among mothers with the introduction of the father’s quota would be negligible. However, adverse effects may become apparent in specific subpopulations, including for couples only entitled to flat-rate benefits (that may therefore need to return to work early), as well as individuals with job insecurity or who are self-employed. Identifying adverse health effects in population subgroups will not only shed light on the differential impacts of parental leave reforms within a population but also will have implications for policy transferability in international contexts where parental leave is generally shorter and discussions on reserved days for the father are ongoing.

The adverse unintended consequences of the reserved days for the mother could to some extent be compensated for by the introduction of the subsequent double days reform, which allowed parents to use a month of parental leave days simultaneously in the crucial first year after birth. In fact, this reform has been shown to have beneficial psychiatric effects for mothers by allowing fathers to participate in childcare as their partners recover from pregnancy and childbirth.^16^

### Theoretical framework

**Figure 1:**
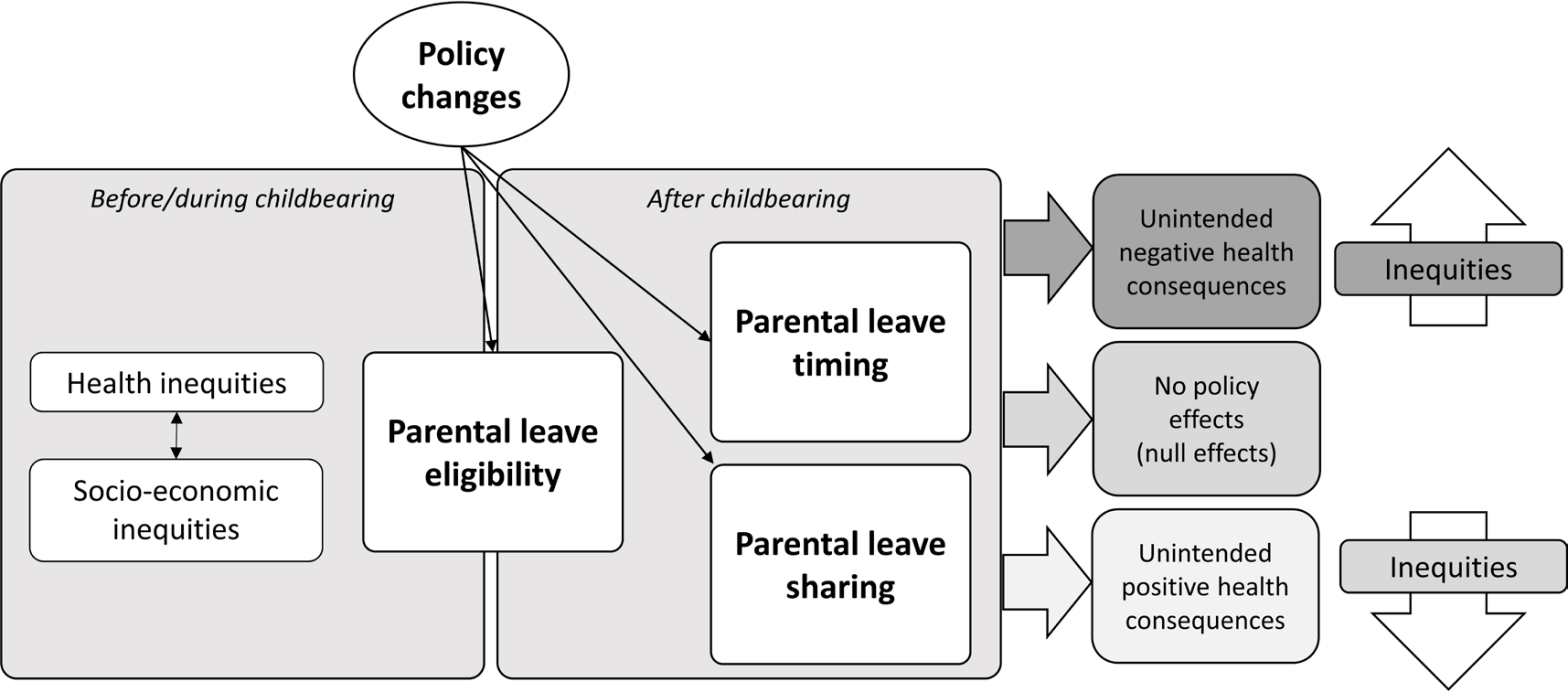
Theoretical framework to study the unintended consequences of parental leave policy from a health equity perspective.

This project is rooted in the Health in All Policies (HiAP) framework, which advocates for considering the health implications of policies across all sectors.^29^ The HiAP approach will help to shed light on the health consequences of a family policy that does not target health, but could have considerable health impacts in society. This approach also highlights how advantages derived from parental leave uptake can be restricted for individuals with poor health, due to the specific work-related requirements for earnings based benefits. Therefore, in this project, health is considered in the sociological perspective of ‘unanticipated (or unintended) consequences of social actions’,^30^ according to which policies and interventions can have latent functional or dysfunctional effects that were not planned or anticipated by relevant actors.^31^ This unexpected dimension reveals the complexity of organised social actions, calling for the investigation of well-intentioned interventions to identify non-linear mechanisms through which such consequences arise (see figure 1). Within this framework, the persistence of (or increase in) health inequalities associated with parental leave policy can be seen as an adverse side effect to be identified and addressed through policy reform.^32^

### Aim/objectives and research questions (RQ)

The aim of this project is to evaluate the consequences of parental leave policy in Sweden from a health equity perspective. More specifically, the project will cover the following RQ:

RQ#1: Is eligibility for earnings based parental leave benefits associated with parents’ mental health?

RQ#2: Is short birth spacing in relation to the speed premium supplement reform associated with health problems in the subsequent pregnancy of the mother (early pregnancy obesity) and the subsequent child (low birth weight, preterm and small-for-gestational age)?

RQ#3: Does the introduction and expansion of the father’s quota reserve days influence the mental health of parents after the birth of their child?

RQ#4: Does the introduction of simultaneous parental leave use through the double days reform influence parents’ mental health?

RQ#5: Does the introduction of the father’s quota and double days reforms influence children’s health (episodes of respiratory tract infections, childhood obesity) or health-related outcomes (duration of breast feeding)?

## Methods and analysis

### Study population

We will include all Swedish-born and foreign-born individuals registered in Sweden between 1973 and 2019 and their Swedish-born children, followed through their first 5 years of life. For most studies, we will focus on a subsample consisting of all women (and their partners) residing in Sweden who had their first birth in the country between 1973 and 2019.

Studies will be primarily focused on primiparous couples, with secondary analyses for multiparous couples. We will only include biological parents, since we aim to examine joint biological and psychosocial consequences of parental leave uptake, with singleton children, given that parents of multiple births are entitled to more days of leave.^6^ Separate analyses by parents’ sociodemographic characteristics, nativity and cohabitation status will be conducted to investigate the heterogeneity of effects in the population.

### Data

**Figure 2:**
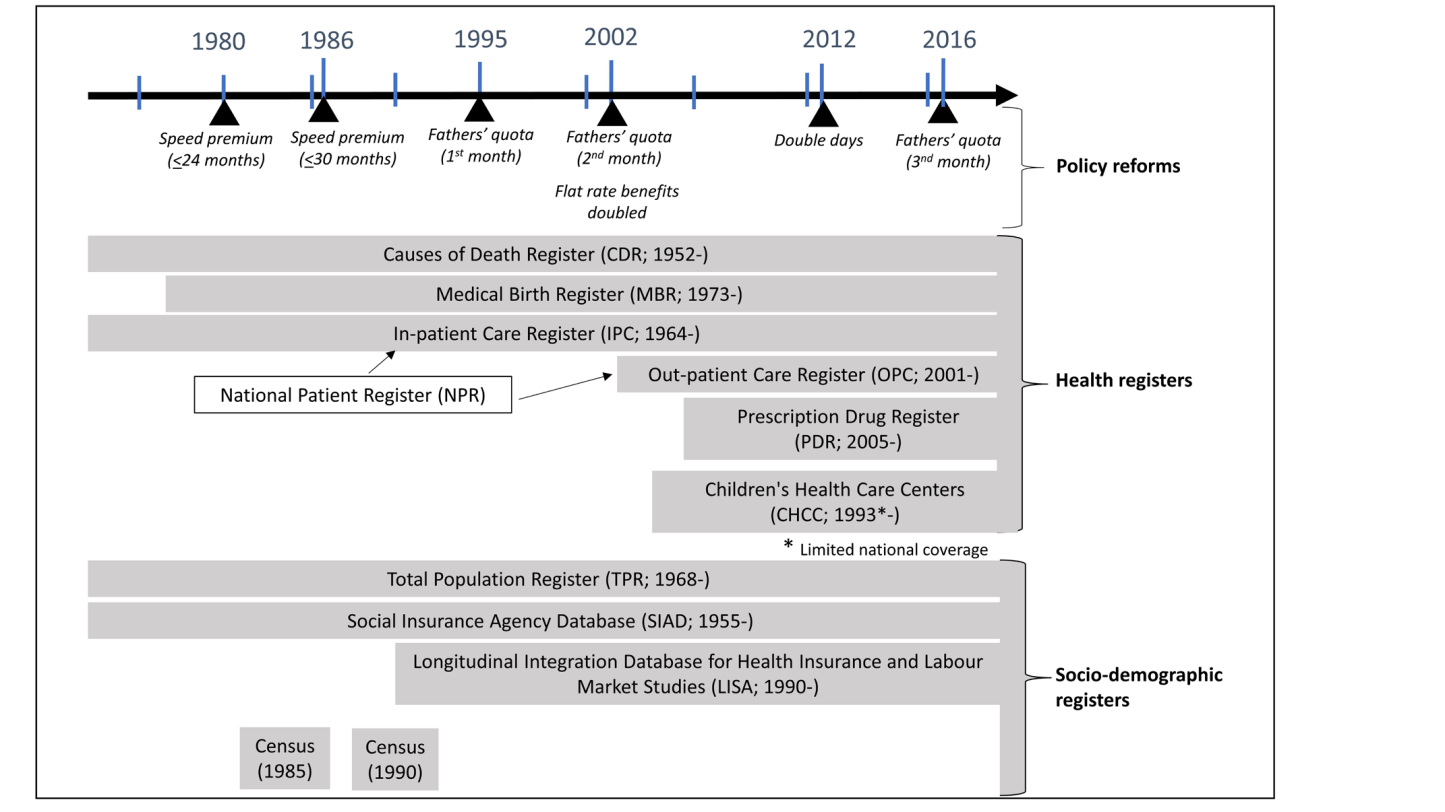
National registers and policy reforms included in the study.

This project will use data from available national registers, collected and linked by the Swedish authorities. Using a unique personal identity number,^33^ we will identify and reconstruct families in the study population through the total population register (TPR; 1968-)^34^ and the multigenerational register (2001-).^35^ Sociodemographic information will be retrieved from the TPR, the Longitudinal Integration Database for Health Insurance and Labour Market Studies (LISA; 1990-),^36^ the Swedish Social Insurance Agency Database (SIAD; 1955-) and the 1985 and 1990 Population and Housing Censuses. We will link health information from the National Patient Register (NPR), on inpatient (IPR; 1964-)^37^ and outpatient care (IPR; 2001-), and the medical birth (MBR; 1973-),^38^ cause of death (1952-)^39^ and prescribed drug (2005-) registers.^40^ We also plan to include information from the Swedish children’s health centres (1993-), with full coverage of longitudinal childhood obesity and breastfeeding data from the counties of Uppsala and Örebro (see figure 2).

### Outcomes

Child health outcomes include low birth weight (<2500 g, irrespective of gestational age), preterm birth (<37 gestational weeks) and a small-for-gestational age measure (<10th percentile of birth weight for each gestational age, based on sex-specific Swedish reference curve for normal foetal growth)^41^ from the MBR; hospitalisation episodes of respiratory tract infections during the first 2 years of life (International Classification of Diseases-10 (ICD-10) Codes J20.5, J21.0, J21.9, J20.9, J18.9, J12.9 and J22.9) from the NPR and duration of partial or exclusive breast feeding (at months 2, 4, 6, 9 and 12) as well as childhood obesity (body mass index (BMI); weight in kilograms/height in meters^2^ using age-specific and sex-specific cut-offs for children’s overweight and obesity^42 43^ at 18 months and 3–5 years) from the CHR. Maternal health outcomes include maternal early pregnancy overweight (BMI 25–29.9 kg/m^2^) and obesity (BMI 30–39.9 kg/m^2^).^44^

Mental health outcomes will be assessed for both parents using count data on purchases of prescription anxiolytic and antidepressant medications (Anatomical Therapeutic Classification Codes N05B and N06A, respectively) as well as outpatient visits and hospitalisations with a primary diagnosis of mental and behavioural disorders (until 1997, ICD-9 Codes 290–319; thereafter, ICD-10 Codes F00-F99), from childbirth to 18 and 36 months after birth.

Outcomes will be expressed as prevalence or rates, given availability of follow-up time.

### Covariates

Annual information on parental leave uptake (number of days) for each parent will also be available from LISA. Specific information on parental leave eligibility and uptake (including length of paid and unpaid leave) will be retrieved from the SIAD.

Demographic characteristics will include parents’ age at childbirth, country of birth (ie, Swedish vs other and specific origins) and marital and cohabitation status, from the TPR and LISA. Sociodemographic characteristics including educational attainment, household disposable income, individual labour income, employment status and receipt of sick leave benefits will be drawn from LISA.

### Study design and statistical analyses

A variety of advanced designs and statistical methods for observational data will be applied. First, using descriptive and correlational designs, we will implement standard multivariable methods to quantify links between prebirth health conditions, parental leave eligibility and uptake, and postbirth health outcomes while accounting for confounding using relevant covariates and heterogeneity analyses.

Second, we will use robust quasi-experimental approaches to assess the causal effects of parental leave policy on health, reducing the risk of selection effects and unmeasured confounding by harnessing the natural, ‘as-if’ randomisation around policies.^45 46^ With this approach, we can isolate the absolute policy effects on health even in the absence of detailed information on changes in parental leave uptake. These approaches include interrupted time series (ITS), regression discontinuity (RD) and difference-in-difference (DiD) designs. For ITS designs, segmented regression models will be adjusted for seasonality and trend while accounting for autocorrelation.^46^ For RD designs, continuity around the cut-off will be tested using the McCrary Density Test and similarity of eligible and ineligible observations will be determined through descriptive statistics. Finally, for DiD designs, we will examine the data to confirm parallel trends and other relevant assumptions.^45^

RQ#1: We will examine the effects of the 2002 doubling of flat-rate benefits on the mental health of parents. An RD design will be applied to plot psychiatric hospitalisation rates with labour income as a forcing variable, with separate plots before (with an income cut-off equivalent to 75 SEK, at 80% reimbursement rate) and after 2002 (with a cut-off equivalent to 150 SEK daily). Cut-offs for both parents’ income will be examined. We will triangulate our findings with a DiD approach, comparing those eligible for the increased benefits to those ineligible before and after reforms.

RQ#2: A segmented-regression ITS of monthly birth data comparing the rate of low birth weight and preterm births 3 years before and after the 1980 and 1986 speed premium reforms will be implemented. We will also compare the prevalence of obesity in mothers early in their second pregnancy before and after the reform.

RQ#3: We will conduct an ITS analysis of parents of children born before and after the 1995 father’s quota reform using segmented regression to capture changes in psychiatric hospitalisation rates up to 36 months after childbirth, based on fathers’ eligibility for the father’s quota.

RQ#4: An ITS for parents to children born before and after the 2012 double days reform will be implemented to capture changes in the number of outpatient visits for mental health and psychotropic prescriptions within 36 months after childbirth, by child’s month of birth.

RQ#5: We will implement separate ITS analyses of children born before and after the 1995 father’s quota and 2012 double days reforms, comparing hospitalisation rates for diagnoses of respiratory tract infections (in children under 2), prevalence of childhood obesity (at 18 months and 3–5 years) and changes in breastfeeding duration.

### Additional analyses

Given the potential for effect modification and compositional changes in the study populations, subgroup analyses by parents’ sociodemographic characteristics (ie, annual labour income, to determine eligibility for earnings based benefits or educational attainment) will be considered for all RQ. Analyses by parents’ nativity will be implemented for RQ#2–4, given differential responses to the policies by Swedish-born and foreign-born parents.^14^ Other relevant parental characteristics, that is, age at childbirth and cohabitation status, will also be considered.

Sensitivity analyses will be performed to account for systematically missing data, such as maternal weight (ie, given that women appearing to be of normal weight are less monitored for obesity).^47^ For studies examining first births, sensitivity analyses restricting mental health outcomes to the 18 months post birth will be considered in order to exclude the potential health effects related to a second pregnancy.

For all ITS designs, we will specify multiple intervention points to assess the possibility of lagged policy effects. Robustness analyses of falsification (‘pseudo-intervention’) dates and falsification outcomes (eg, prebirth hospitalisations) will also be considered.

### Limitations

Despite our reliance on a robust quasi-experimental approach, it is difficult to predict the relative timing of the behavioural response to each parental leave reform (ie, in uptake) and its health consequences. Parents have been shown to respond quickly to policy changes,^14 15 27^ but given that some parents may not be aware of their eligibility on the reform dates, delayed reaction times could lead to lagged health effects for subgroups such as migrants.^12^ Thus, we will use a long follow-up both within the life course (ie, years since birth) and in the context of the policy (ie, years since reform implemented).

Quasi-experimental designs have the potential to reduce unmeasured confounding, but policies themselves, as well as ongoing trends, could inadvertently modify the composition of the study populations, potentially biasing any causal interpretations. For one, the speed premium may have affected the composition of individuals who decided to have children after the reform, that is, encouraging more parents eligible for earnings based benefits to have children after 1986, given their increased level of benefits for subsequent children. Exogenous impacts other than the policy under investigation could also contribute to changing population compositions, as in the case of the 1990s economic crisis, which may have discouraged couples with lower socioeconomic position from having children after the 1995 father’s quota. We will thus examine varying sociodemographic characteristics of the study populations before and after each reform to assess the robustness of the design to approximate causal effects, with corresponding subgroup and sensitivity analyses.

Registers generally have greater coverage than other data sources but may be prone to bias in the form of missing data and overcoverage (ie, unreported outmigration), which we will address in appropriate sensitivity analyses. The historical availability of register data may also influence our analytical choices. For example, to evaluate the impact of the father’s quota on psychiatric health, we will primarily look at the first reserved month introduced in 1995.^6^ However, only severe health outcomes (psychiatric hospitalisations) can be assessed with the available registers at that time. In order to evaluate less severe outcomes (outpatient visit and prescription data) of increased fathers’ leave use, we will evaluate the more recent double days reform.^16^

Finally, although our primary aim is to evaluate unintended adverse health effects of these parental leave reforms, we will also consider health benefits. For example, short birth intervals promoted by the speed premium supplement could also have led to women having subsequent children at younger age, thereby reducing the risk for adverse reproductive outcomes associated with advanced age (eg, foetal abnormalities, preterm and stillbirths).^48–50^

By considering both negative and positive health outcomes, in conjunction with a variety of parental leave reforms and parental characteristics, this study will openly explore future directions for international parental leave research.

### Patient and public involvement

This project will not involve any patients or the public. However, we will attempt to communicate the findings of the study to the public through the mass media.

## Ethics and dissemination

Although we handle personal information, individual consent is not required since the information is anonymised by the agencies responsible for data protection, and none of the records will have personal identifiers attached. Statistics Sweden may also aggregate some information (such as country of birth) to ensure that individuals cannot be identified in relation to other variables (eg, education or place of residence). Nevertheless, since our dataset contains individual-level information and thus requires special provisions for its use, we applied for all necessary ethical permissions from the Stockholm Regional Ethical Review Board to cover the specific RQ addressed by this project (Dnr 2019-04913).

## Supplementary Material

Reviewer comments

Author's manuscript

## Data Availability

Data may be obtained from a third party and are not publicly available. This study is a protocol that plan to be conducted using data from Swedish registers. Studies will be conducted under the Swedish Statistics Act, where privacy concerns restrict the availability of register data for research. In order to access Swedish register data, researchers must contact the agencies responsible for data protection, conditional on ethical vetting.
