# Supplementary material for: Unintended health consequences of Swedish parental leave policy (ParLeHealth): protocol for a quasi-experimental study
Source: BMJ Open. 2021 Jun 9;11(6):e049682. doi: 10.1136/bmjopen-2021-049682 (PMC8191630; doi:10.1136/bmjopen-2021-049682)
Supplement: Author's manuscript [file bmjopen-2021-049682.draft_revisions.pdf]

BMJ Open is committed to open peer review. As part of this commitment we make the peer review history of every article we publish publicly available.

When an article is published we post the peer reviewers' comments and the authors' responses online. We also post the versions of the paper that were used during peer review. These are the versions that the peer review comments apply to.

The versions of the paper that follow are the versions that were submitted during the peer review process. They are not the versions of record or the final published versions. They should not be cited or distributed as the published version of this manuscript.

BMJ Open is an open access journal and the full, final, typeset and author-corrected version of record of the manuscript is available on our site with no access controls, subscription charges or pay-per-view fees (http://bmjopen.bmj.com).

If you have any questions on BMJ Open's open peer review process please email info.bmjopen@bmj.com

BMJ Open

Unintended health consequences of Swedish parental leave policy (ParLeHealth): Protocol for a quasi-experimental study

Journal:	*BMJ Open*
Manuscript ID	bmjopen-2021-049682
Article Type:	Protocol
Date Submitted by the Author:	30-Jan-2021
Complete List of Authors:	Juárez , Sol Pia; Stockholm University, Department of Public Health Sciences; Centre for Health Equity Studies, Stockholm University/Karolinska Institute<br>Honkaniemi, Helena ; Stockholm University,<br>Heshmati, Amy; Stockholm University, Department of Public Health Sciences; Karolinska Institute, Department of Global Public Health<br>Debiasi, Enrico; Stockholms Universitet, Department of Public Health Sciences<br>Dunlavy, Andrea; Stockholm University, Department of Public Health Sciences<br>Hjern, Anders; Stockholm University, Centre for Health Equity Studies<br>Rostila, Mikael ; Stockholm University, Department of Public Health Sciences<br>Mussino, Eleonora; Stockholms Universitet, Stockholm University Demography Unit<br>Katikireddi, Srinivasa; University of Glasgow, MRC/CSO Social & Public Health Sciences Unit<br>Duvander, Ann-Zofie; Stockholm University, Demographic Unit (SUDA); Mid University, Department of Sociology
Keywords:	MENTAL HEALTH, Child protection < PAEDIATRICS, PUBLIC HEALTH, REPRODUCTIVE MEDICINE

SCHOLARONE™
Manuscripts

I, the Submitting Author has the right to grant and does grant on behalf of all authors of the Work (as defined in the below author licence), an exclusive licence and/or a non-exclusive licence for contributions from authors who are: i) UK Crown employees; ii) where BMJ has agreed a CC-BY licence shall apply, and/or iii) in accordance with the terms applicable for US Federal Government officers or employees acting as part of their official duties; on a worldwide, perpetual, irrevocable, royalty-free basis to BMJ Publishing Group Ltd ("BMJ") its licensees and where the relevant Journal is co-owned by BMJ to the co-owners of the Journal, to publish the Work in this journal and any other BMJ products and to exploit all rights, as set out in our licence.

The Submitting Author accepts and understands that any supply made under these terms is made by BMJ to the Submitting Author unless you are acting as an employee on behalf of your employer or a postgraduate student of an affiliated institution which is paying any applicable article publishing charge ("APC") for Open Access articles. Where the Submitting Author wishes to make the Work available on an Open Access basis (and intends to pay the relevant APC), the terms of reuse of such Open Access shall be governed by a Creative Commons licence – details of these licences and which Creative Commons licence will apply to this Work are set out in our licence referred to above.

Other than as permitted in any relevant BMJ Author's Self Archiving Policies, I confirm this Work has not been accepted for publication elsewhere, is not being considered for publication elsewhere and does not duplicate material already published. I confirm all authors consent to publication of this Work and authorise the granting of this licence.

For peer review only

Unintended health consequences of Swedish parental leave policy (ParLeHealth): Protocol for a quasi-experimental study

Sol P. Juárez[1,2*] sol.juarez@su.se, ORCID iD: 0000-0001-9086-7588

Helena Honkaniemi[1,2] helena.honkaniemi@su.se, ORCID iD: 0000-0003-0800-0892

Amy Heshmati[1,2,3] amy.heshmati@su.se, ORCID iD: 0000-0002-1462-7328

Enrico Debiasi[1,2] enrico.debiasi@su.se, ORCID iD:0000-0001-5772-5510

Andrea Dunlavy[1,2] andrea.dunlavy@su.se, ORCID iD: 0000-0003-4661-3462

Anders Hjern[1,2,4] anders.hjern@su.se, ORCID iD: 0000-0002-1645-2058

Mikael Rostila[1,2] mikael.rostila@su.se, ORCID iD: 0000-0002-6973-0381

Eleonora Mussino[5] eleonora.mussino@sociology.su.se, ORCID iD: 0000-0002-5311-4277

Srinivasa Vittal Katikireddi[1,2,6] vittal.katikireddi@glasgow.ac.uk, ORCID iD: 0000-0001-6593-9092

Ann-Zofie Duvander[5,7] ann-zofie.duvander@sociology.su.se, ORCID iD: 0000-0001-5241-4588

[1] Department of Public Health Sciences, Stockholm University, Stockholm, Sweden.

[2] Centre for Health Equity Studies, Stockholm University/Karolinska Institutet, Stockholm, Sweden.

[3] Department of Global Public Health, Karolinska Institutet, Stockholm, Sweden.

[4] Department of Medicine, Karolinska Institutet, Stockholm, Sweden

[5] Demographic Unit (SUDA), Department of Sociology, Stockholm University, Stockholm, Sweden.

[6] Institute of Health & Wellbeing. University of Glasgow

[7] Department of Sociology, Mid University, Östersund, Sweden

[*] Corresponding author: Sol P. Juárez, Department of Public Health Sciences, Stockholm University, SE-106 91, Stockholm, Sweden

For peer review only

Word count: 3,972 (up to 4000)
Abstract: 298 (up to 300)
Illustrations: 2
References: 48

ABSTRACT

Introduction: Sweden has long been praised for a generous parental leave policy oriented towards facilitating a gender-equitable approach to work and parenting. Yet certain aspects of Swedish parental leave could also be responsible for the maintenance of (or even the increase in) health inequalities. Using a 'Health in All Policies' lens, this research project aims to assess the unintended health consequences of various components of Sweden's parental leave policy, including eligibility for and uptake of earnings-based benefits.

Methods and analysis: We will utilise individual-level data from multiple Swedish registers. Sociodemographic information, including on parental leave use, will be retrieved from the Total Population Register, Longitudinal Integration Database for Health Insurance and Labour Market Studies, and Social Insurance Agency Registers. Health information for parents and children will be retrieved from the Patient, Prescribed Drug, Cause of Death, Medical Birth and Children's Health Registers. We will evaluate parents' mental, mothers' reproductive and children's general health outcomes in relation to several policy reforms aiming to protect parental leave benefits in short birth spacing (the *speed premium*) and to promote father's uptake (the *Father's quota*) and sharing of parental leave days (the *Double days* reform). We will also examine effects of increases in basic parental leave benefit levels. Using quasi-experimental designs, we will compare health outcomes across these reforms and eligibility thresholds with interrupted time series, difference-in-difference, and regression discontinuity approaches to reduce the risk of health selection and assess causality in the link between parental leave use and health.

Ethics and dissemination: This project has been granted all necessary ethical permissions from the Stockholm Regional Ethical Review Board (Dnr 2019-04913) for accessing and analysing de-identified data. The final outputs will primarily be disseminated as scientific articles published in open-access, high-impact peer-reviewed international journals, as well as press releases and policy briefs.

For peer review only

For peer review only - http://bmjopen.bmj.com/site/about/guidelines.xhtml

STRENGTHS AND LIMITATIONS OF THIS STUDY

- This will be the first project to evaluate multiple aspects of Swedish parental leave policy by adopting a health equity perspective and Health in All Policies (HiAP) approach.
- The study will utilise quasi-experimental methods on total population register data to assess causal links between parental leave uptake and health.
- The study will expand on existing evidence of health effects by considering outcomes not only for mothers and children, but fathers as well.
- Analyses will be restricted to studying health outcomes that are available within administrative datasets, thereby precluding assessment of potentially more sensitive self-reported assessments of health.

For peer review only

For peer review only - http://bmjopen.bmj.com/site/about/guidelines.xhtml

INTRODUCTION

Childbearing is a life event with significant social and health implications for parents, children and society alike. Among women, childbearing has been considered to be responsible for lower labour market participation and gendered wage gaps (i.e., the 'motherhood penalty'),[1] resulting in lower income protection in old age.[1-3] Childbearing is also a risk factor for various physical and mental health conditions among mothers.[4] By offering secure time off work for childcare and recovery, parental leave has the potential to reduce social and health inequalities in society. However, given that parental leave eligibility and uptake may vary across social strata, it may also have the potential to (re)produce social inequalities in health.

1. The Swedish parental leave system

We report intervention details according to the TIDieR-PHP reporting guidelines.[5]

1.1 Length and flexibility of parental leave

Sweden has developed into one of the most generous and flexible paid parental leave systems worldwide.[6] Starting in 1974, parents were entitled to six months of leave to be used within 270 days of birth and shared by the parents as they desired. An additional nine months of leave were introduced incrementally from 1975 to 1989,[6,7] with an extension to use a portion of the leave until the child turned eight years old in 1978. In 1995, one month of the existing length of leave was reserved for each parent, which was further extended by one month each in 2002 and 2016.[8,9] Since 2014, couples have been entitled to use 480 parental leave days up until the child turns 12 years old, but with no more than 96 days from the child's fourth birthday.[7]

We will focus on mothers' and fathers' *paid parental leave* (i.e., job-protected leave paid at set benefit levels) rather than *unpaid parental leave* (i.e., job-protected unpaid leave in the first 18 months after birth). Furthermore, we do not examine *maternity leave* (i.e., two weeks of mandatory job-protected leave for the mother prior to or after childbirth, which can be unpaid

For peer review only - http://bmjopen.bmj.com/site/about/guidelines.xhtml

or paid as standard parental leave days), *temporary leave*, including at birth/adoption (formerly *paternity leave*; i.e., up to ten days of separate paid, job-protected leave for partners to attend the delivery or assist the mother with infant care), or leave for the care of sick children.[6,7]

1.2 Parental leave eligibility and compensation

In Sweden, all legally residing parents are eligible to receive parental leave benefits (i.e., job-protected leave), including biological parents as well as individuals with child custody.[10] Individuals must apply for these benefits through the Social Insurance Agency, the governmental authority that decides on and administers insurance and contributions to families and children in Sweden.[11] The benefits are split into general flat-rate or earnings-based benefits, collectively referred to as 'parental benefits' and applied to 390 out of the total 480 days, while the remaining 90 days are paid at a second flat rate, henceforth referred to as 'guarantee days'.[7]

To be eligible for earnings-based parental benefits, an individual must have been employed for at least 240 consecutive days prior to the estimated delivery date with a minimum annual income (82,100 Swedish kronor (SEK) in 2020). Although the remuneration level has varied over time, individuals today receive approximately 80% of their salary up to a certain ceiling (1,006 SEK per day in 2020) from the Social Insurance Agency, with potential top-ups from the employer.[12] If an individual has an income below a certain level (117,590 SEK in 2020) during the 240 days before childbirth, they are only entitled to a general flat-rate benefit (250 SEK per day in 2020) for the first 180 days which they claim for the child. After 180 claimed days, parents can receive benefits based on their current earnings.[7] Finally, if an individual has no income or an annual income below the stipulated level throughout the leave period they will receive the general flat-rate benefit for the full 390 days. However, if an individual is sick, a student, or unemployed but a 'job seeker', they can receive parental leave benefits based on their prior income if they have registered with the Public Employment Service.[10] Regardless of

For peer review only - http://bmjopen.bmj.com/site/about/guidelines.xhtml

employment history, the final 90 days are always paid at a minimum guaranteed level, paid at 60 SEK starting in 1987, and 180 SEK since 2006.[7]

1.2 Policy reforms

The Swedish parental leave system has undergone several reforms over the past half-century. We define a 'policy reform' as any change implemented at a specific time that could impact either uptake or sharing of parental leave, as well as eligibility for earnings-based benefits. To evaluate the unforeseen health consequences of the parental leave policy – i.e., a welfare policy that *does not* primarily target health – we will concentrate on reforms that have either been shown to accomplish their primary aim (i.e., the *speed premium*, *Father's quota*, and *Double days* reforms) or with undoubtedly significant, albeit unintended, impacts (i.e., the doubled value of general flat-rate benefits).

Doubled flat-rate benefits: Although not explicitly a reform, the doubling of the general flat-rate benefits from 60 SEK per day (from 1987) to 120 SEK per day (in 2002) aimed to secure the economic welfare of low-income families.

Speed premium: Since earnings-related parental leave benefits are estimated based on one's salary prior to childbearing (at approximately 80% of the salary), parents having children in quick succession may choose to temporarily reduce their working hours or be unable to return to work at all, and will thus have lower benefits for their second and subsequent births. In 1980, a supplement (commonly referred to as the "speed premium") was introduced to safeguard women's income between parental leave periods, ensuring that for births with up to 24 months' spacing,[13] and then up to 30 months' spacing (following a 1986 expansion),[14] all earnings-based benefits could be based on the parents' salaries prior to the birth of their previous child, if higher than their current income. An evaluation of the 1986 "speed premium" expansion

For peer review only - http://bmjopen.bmj.com/site/about/guidelines.xhtml

revealed that an increased proportion of Swedish-born parents had children in short succession; therefore it has been called the speed premium, even if this was an unintended consequence.[14]

Father's quota: Fathers have been eligible to use parental leave since 1974, when parents could choose to divide parental leave days between themselves as they saw fit. However, most fathers' days were transferred to and claimed by mothers, with an average use of 320 days in 1994, reinforcing mothers' "double burden" of childcare and work. In response, a *Father's quota* reform was implemented in 1995, reserving a month of leave exclusively for the father, to be forfeited completely if left unused (similarly, a 30-day reserve was allocated to the mother, i.e., a *Mother's quota*). It succeeded in increasing the proportion of fathers using parental leave from 43% to 75%.[15] The *quota* was expanded to two and three months in 2002 and 2016, respectively, but with smaller impacts on uptake.[6]

Double days: In 2012, 30 days of existing parental leave were designated as 'shareable', i.e., could be used simultaneously by both parents within the child's first year of life. By granting flexibility to the couple in their leave use, this "Double days" reform aimed to facilitate the transfer of care from one parent to another. In fact, the reform significantly increased the proportion and number of father's leave days used in the first half-year after childbirth.[16]

All reforms were implemented nationally on January 1st of the indicated year.

2. Unintended health consequences of the Swedish parental leave system

Despite the relative generosity of the Swedish parental leave system and its efforts to promote gender equity, some aspects could be responsible for the maintenance or increase in social and health inequities. For example, the strong work-eligibility requirement for earnings-based parental leave benefits may exclude groups with less stability in the labour market, such as students and migrants, from benefits equal to those enjoyed by persons with stable incomes.[17]

For peer review only - http://bmjopen.bmj.com/site/about/guidelines.xhtml

Inequities in the work-eligibility requirement can emerge in various ways, either by restricting the parental leave benefits scheme to the securely employed and coincidentally excluding parents with ill health (i.e., *health selection*), or by creating health inequities through the unequal distribution of benefits (i.e., *social causation*). For example, individuals with long-term illness may be more likely to have a weak labour market attachment, which in turn can exclude them from earnings-related parental leave benefits, thus further increasing socioeconomic inequities. Based on this, we expect that the *doubled flat-rate benefits* could increase the uptake among lower income families or decrease the economic trade-off of using parental leave among lower income families, thus having potential psychosocial benefits.

Parental leave policies might impact health differently following certain policy reforms. For example, the *speed premium* supplement introduced to secure earnings-based parental leave benefits to parents who have children in quick succession essentially incentivises short birth spacing.[14] This might lead to increased psychosocial stress and adverse reproductive outcomes among women who become pregnant whilst simultaneously rearing an infant or toddler. Short birth intervals (defined as consecutive births within 33 months, assuming nine months of gestation)[18] have been associated with nutrition depletion, as the mother might not have enough time between births to recover her nutritional reserves,[14] and higher risks of weight gain and obesity throughout life.[15] In turn, these issues are risk factors for adverse outcomes in subsequent children, including low birthweight, preterm birth and perinatal death.[19]

The *Father's quota* was implemented to promote a gender-equitable work-life balance and shared parenting. However, the health consequences of the *Father's quota* have been debated, with a lack of empirical evidence on its differing mechanisms. Studies have shown a positive association between the father's parental leave use and healthy behaviours, including decreased

For peer review only - http://bmjopen.bmj.com/site/about/guidelines.xhtml

alcohol use and increased physical activity,[20,21] as well as decreased mortality risks.[22] Nonetheless, there may be situations in which fathers' leave use is less optimal for or even unfavourable for the family. The quota may have encouraged mothers to return to work sooner, potentially interrupting their physical and psychological recovery from pregnancy and childbirth. These potential adverse effects among parents could extend to the child: shorter maternity leave in the child's first months could shorten breastfeeding, a risk factor for childhood obesity[23] and respiratory track infectious in the first two years of life.[24] However, there is a lack of evidence on changes in mothers' labour supply following the reform,[25,26] suggesting that mothers may have stayed at home with fathers on an unpaid basis, foregoing their income and thus potentially creating psychological strain, especially among low-income parents.

Given that parental leave in Sweden is longer than in most other contexts, it can be argued that any health effect of shortened parental leave among mothers with the introduction of the *Father's quota* would be negligible. However, adverse effects may become apparent in specific sub-populations, including for couples only entitled to flat-rate benefits (that may therefore need to return to work early), as well as individuals with job insecurity or who are self-employed. Identifying adverse health effects in population sub-groups will not only shed light on the differential impacts of parental leave reforms within a population but will have implications for policy transferability in international contexts where parental leave is generally shorter and discussions on reserved days for the father are ongoing.

The adverse unintended consequences of the reserved days for the mother could to some extent be compensated for by the introduction of the subsequent *Double days* reform, which allowed parents to use a month of parental leave days *simultaneously* in the crucial first year after birth.

For peer review only - http://bmjopen.bmj.com/site/about/guidelines.xhtml

In fact, this reform has been shown to have beneficial psychiatric effects for mothers by allowing fathers to participate in childcare as their partners recover from pregnancy and childbirth.[16]

3. *Theoretical framework*

This project is rooted in the Health in All Policies (HiAP) framework, which advocates for considering the health implications of policies across all sectors.[27] The HiAP approach will help to shed light on the health consequences of a family policy that does not target health, but could have considerable health impacts in society. This approach also highlights how advantages derived from parental leave uptake can be restricted for individuals with poor health, due to the specific work-related requirements for earnings-based benefits. Therefore, in this project health is considered in the sociological perspective of 'unanticipated (or unintended) consequences of social actions', [28] according to which policies and interventions can have latent functional or dysfunctional effects that were not planned or anticipated by relevant actors.[29] This unexpected dimension reveals the complexity of organised social actions, calling for the investigation of well-intentioned interventions to identify non-linear mechanisms through which such consequences arise (see **Figure 1**). Within this framework, the persistence of (or increase in) health inequalities associated with parental leave policy can be seen as an adverse side effect to be identified and addressed through policy reform.[30]

4. *Aim/objectives and research questions*

The aim of this project is to evaluate the consequences of parental leave policy in Sweden from a health equity perspective. More specifically, the project will cover the following research questions (RQ):

For peer review only - http://bmjopen.bmj.com/site/about/guidelines.xhtml

RQ#1. Is eligibility for earnings-based parental leave benefits associated with parents' mental health?

RQ#2. Is short birth spacing in relation to the *speed premium supplement reform* associated with health problems in the subsequent pregnancy of the mother (early pregnancy obesity) and the subsequent child (low birthweight, preterm and small-for-gestational age)?

RQ#3. Does the introduction and expansion of the *Father's quota* reserve days influence the mental health of parents after the birth of their child?

RQ#4. Does the introduction of simultaneous parental leave use through the *Double days* reform influence parents' mental health?

RQ#5: Does the introduction of the *Father's quota* and *Double days* reforms influence children's health (episodes of respiratory tract infections, childhood obesity) or health-related outcomes (duration of breastfeeding)?

METHODS AND ANALYSIS

Study population

We will include all Swedish- and foreign-born individuals registered in Sweden between 1973 and 2019 and their Swedish-born children, followed through their first five years of life. For most studies, we will focus on a subsample consisting of all women (and their partners) residing in Sweden who had their first birth between 1973 and 2019.

Studies will be primarily focused on primiparous couples, with secondary analyses for multiparous couples. We will only include biological parents, since we aim to examine joint biological and psychosocial consequences of parental leave uptake, with singleton children, given that parents of multiple births are entitled to more days of leave.[6] Separate analyses by

For peer review

parents' sociodemographic characteristics, nativity, and cohabitation status will be conducted to investigate the heterogeneity of effects in the population.

Data

This project will use data from available national registers, collected and linked by the Swedish authorities. Using a unique personal identity number,[31] we will identify and reconstruct families in the study population through the Total Population Register (TPR; 1968-)[32] and the Multigenerational Register (MGR; 2001-).[33] Sociodemographic information will be retrieved from the TPR, the Longitudinal Integration Database for Health Insurance and Labour Market Studies (LISA; 1990-),[34] the Swedish Social Insurance Agency Database (SIAD; 1955-) and the 1985 and 1990 Population and Housing Censuses. We will link health information from the National Patient Register (NPR), on inpatient (IPR; 1964-)[35] and outpatient care (IPR; 2001-); and the Medical Birth (MBR; 1973-),[36] Cause of Death (1952-)[37] and Prescribed Drug (2005-) Registers.[38] We also plan to include information from the Swedish Children's Health Centers (CHC; 1993-), with full coverage of longitudinal childhood obesity and breastfeeding data from the countries of Uppsala and Örebro (see **Figure 2**).

Outcomes

Child health outcomes include low birthweight (<2,500 grams, irrespective of gestational age), preterm birth (<37 gestational weeks), and a small-for-gestational age measure (<10th percentile of birthweight for each gestational age, based on sex-specific Swedish reference curve for normal foetal growth)[39] from the MBR; hospitalisation episodes of respiratory tract infections during the first two years of life (International Classification of Diseases-10 [ICD-10] Codes J20.5, J21.0, J21.9, J20.9, J18.9, J12.9 and J22.9) from the NPR; and duration of

partial or exclusive breastfeeding (at months 2, 4, 6, 9 and 12) as well as childhood obesity (body mass index [BMI; weight in kilograms/height in meters^2] using age- and sex-specific cut-offs for children's overweight and obesity[40,41] at 18 months and 3, 4 and 5 years old) from the CHR. Maternal health outcomes include maternal early pregnancy overweight (BMI 25-29.9 kg/m^2) and obesity (BMI 30-39.9 kg/m^2).[42]

Mental health outcomes will be assessed for both parents using count data on purchases of prescription anxiolytic and antidepressant medications (Anatomical Therapeutic Classification or ATC Codes N05B and N06A, respectively) and hospitalisations with a primary diagnosis of mental and behavioural disorders (until 1997, ICD-9 Codes 290-319; thereafter, ICD-10 Codes F00-F99), from childbirth to 18 and 36 months after birth.

Outcomes will be expressed as prevalence or rates, given availability of follow-up time.

Covariates

Annual information on parental leave uptake (number of days) for each parent will also be available from LISA. Specific information on parental leave eligibility and uptake (including length of paid and unpaid leave) will be retrieved from the SIAD.

Demographic characteristics will include parents' age at childbirth, country of birth (i.e., Swedish vs. other and specific origins), and marital and cohabitation status, from the TPR and LISA. Sociodemographic characteristics including educational attainment, household disposable income, individual labour income, employment status and receipt of sick leave benefits will be drawn from LISA.

Study design and statistical analysis

For peer review only - http://bmjopen.bmj.com/site/about/guidelines.xhtml

A variety of advanced designs and statistical methods for observational data will be applied. First, using descriptive and correlational designs, we will implement standard multivariable methods to quantify links between pre-birth health conditions, parental leave eligibility and uptake, and post-birth health outcomes while accounting for confounding using relevant covariates and heterogeneity analyses.

Second, we will use robust quasi-experimental approaches to assess the causal effects of parental leave policy on health, reducing the risk of selection effects and unmeasured confounding by harnessing the natural, "as-if" randomisation around policies.[43,44] With this approach, we can isolate the absolute policy effects on health even in the absence of detailed information on changes in parental leave uptake. These approaches include interrupted time series (ITS), regression discontinuity (RD) and difference-in-difference (DiD) designs. For ITS designs, segmented regression models will be adjusted for seasonality and trend while accounting for autocorrelation.[44] For RD designs, continuity around the cut-off will be tested using the McCrary density test and similarity of eligible and ineligible observations will be determined through descriptive statistics. Finally, for DiD designs, we will examine the data to confirm parallel trends and other relevant assumptions.[43]

RQ#1: We will examine the effects of the 2002 *doubling of flat-rate benefits* on the mental health of parents. An RD design will be applied to plot psychiatric hospitalisation rates with labour income as a forcing variable, with separate plots before (with an income cut-off equivalent to 75 SEK, at 80% reimbursement rate) and after 2002 (with a cut-off equivalent to 150 SEK daily). Cut-offs for both parents' income will be examined. We will triangulate our findings with a DiD approach, comparing those eligible for the increased benefits to those ineligible before and after reforms.

For peer review only - http://bmjopen.bmj.com/site/about/guidelines.xhtml

RQ#2: A segmented-regression ITS of monthly birth data comparing the rate of low birthweight and preterm births three years before and after the 1986 *speed premium* reform will be implemented. We will also compare the prevalence of obesity in mothers early in their second pregnancy before and after the reform.

RQ#3: We will conduct an ITS analysis of parents of children born before and after the 1995 *Father's quota* reform using segmented regression to capture changes in psychiatric hospitalisation rates up to 36 months after childbirth based on fathers' eligibility for the *Father's quota*.

RQ#4: An ITS for parents to children born before and after the 2012 *Double days* reform will be implemented to capture changes in the number of psychotropic prescriptions and psychiatric hospitalisations within 36 months after childbirth by child's month of birth.

RQ#5: We will implement separate ITS analyses of children born before and after the 1995 *Father's quota* and 2012 *Double days* reforms, comparing hospitalisation rates for diagnoses of respiratory tract infections (in children under 2), prevalence of childhood obesity (at 18 months and 3, 4 and 5 years old), and changes in breastfeeding duration.

Additional analyses

Given the potential for effect modification and compositional changes in the study populations, sub-group analyses by parents' sociodemographic characteristics (i.e., annual labour income, to determine eligibility for earnings-based benefits, or educational attainment) will be considered for all research questions. Analyses by parents' nativity will be implemented for RQ#2-4, given differential responses to the policies by Swedish- and foreign-born parents.[14] Other relevant parental characteristics, i.e., age at childbirth and cohabitation status, will also be considered.

For peer review only

Sensitivity analyses will be performed to account for systematically missing data, such as maternal weight (i.e., given that women appearing to be of normal weight are less monitored for obesity).[45] For studies examining first births, sensitivity analyses restricting mental health outcomes to the 18 months post-birth will be considered in order to exclude the potential health effects related to a second pregnancy.

For all ITS designs, we will specify multiple intervention points to assess the possibility of lagged policy effects. Robustness analyses of falsification ('pseudo-intervention') dates, and falsification outcomes (e.g., pre-birth hospitalisations) will also be considered.

Limitations

Despite our reliance on a robust quasi-experimental approach, it is difficult to predict the relative timing of the behavioural response to each parental leave reform (i.e., in uptake) and its health consequences. Parents have been shown to respond quickly to policy changes,[14,15,25] but given that some parents may not be aware of their eligibility on the reform dates, delayed reaction times could lead to lagged health effects for sub-groups such as migrants.[12] Thus, we will utilise a long follow-up both within the life course (i.e., years since birth) and in the context of the policy (i.e., years since reform implemented).

Quasi-experimental designs have the potential to reduce unmeasured confounding, but policies themselves, as well as ongoing trends, could inadvertently modify the composition of the study populations, potentially biasing any causal interpretations. For one, the *speed premium* may have affected the composition of individuals who decided to have children after the reform, i.e., encouraging more parents eligible for earnings-based benefits to have children after 1986, given their increased level of benefits for subsequent children. Exogenous impacts other than the policy under investigation could also contribute to changing population compositions, as in the case of the 1990s economic crisis, which may have discouraged couples with lower

For peer review only - http://bmjopen.bmj.com/site/about/guidelines.xhtml

socioeconomic position (SEP) from having children after the 1995 *Father's quota*. We will thus examine varying sociodemographic characteristics of the study populations before and after each reform to assess the robustness of the design to approximate causal effects, with corresponding sub-group and sensitivity analyses.

Registers generally have greater coverage than other data sources but may be prone to bias in the form of missing data and over-coverage (i.e., unreported outmigration), which we will address in appropriate sensitivity analyses. The historical availability of register data may also influence our analytical choices. For example, to evaluate the impact of the *Father's quota* on psychiatric health, we will primarily look at the first reserved month introduced in 1995.[6] However, only severe health outcomes (psychiatric hospitalisations) can be assessed with the available registers at that time. In order to evaluate less severe outcomes (prescription data) of increased fathers' leave use, we will evaluate the more recent *Double days* reform.[16]

Finally, although our primary aim is to evaluate unintended adverse health effects of these parental leave reforms, we will also consider health benefits. For example, short birth intervals promoted by the *speed premium* supplement could also have led to women having subsequent children at younger age, thereby reducing the risk for adverse reproductive outcomes associated with advanced age (e.g., foetal abnormalities, preterm and stillbirths).[46-48]

By considering both negative and positive health outcomes, in conjunction with a variety of parental leave reforms and parental characteristics, this study will openly explore future directions for international parental leave research.

For peer review only - http://bmjopen.bmj.com/site/about/guidelines.xhtml

Patient and public involvement

This project will not involve any patients or the public. However, we will attempt to communicate the findings of the study to the public through the mass media.

Ethics and dissemination

Although we handle personal information, individual consent is not required since the information is anonymised by the agencies responsible for data protection, and none of the records will have personal identifiers attached. Statistics Sweden may also aggregate some information (such as country of birth) to ensure that individuals cannot be identified in relation to other variables (e.g., education or place of residence). Nevertheless, since our dataset contains individual-level information and thus requires special provisions for its use, we applied for all necessary ethical permissions from the Stockholm Regional Ethical Review Board to cover the specific research questions addressed by this project (Dnr 2019-04913).

Author's contribution

SPJ, HH and AD conceived and designed the study with feedback from EM and AZD (regarding the policy), MR and AH (regarding health outcomes and data access) and VK (regarding methodology). SPJ drafted the manuscript with help from HH, ED and AMH and all authors made substantial contributions. All authors approved the final version submitted for publication. SPJ is the guarantor.

Funding statement

This work was supported by the Swedish Research Council (VR) grant number 2018-01825 and the Swedish Council for Health, Working Life, and Social welfare (FORTE) Grant number 2016–07128. In addition, SVK acknowledges funding from a NRS Senior Clinical fellowship (SCAF/15/02), the Medical Research Council (MC_UU_12017/13) and the Scottish Government Chief Scientist Office (SPHSU13).

Funders of the study had no involvement in study design and will not be involved in any parts of the study, including data collection, data analysis, data interpretation, or writing of the report.

Competing Interests Statement: None

For peer review only

REFERENCES

1. Budig MJ, England P. The wage penalty for motherhood. *Am Sociol Rev.* 2001;66(2):204-225.
2. England P. Gender inequality in labor markets: The role of motherhood and segregation. *Social Politics: International Studies in Gender, State & Society.* 2005;12(2):264-288.
3. Charles M. A world of difference: international trends in women's economic status. *Annu Rev Sociol.* 2011;37:355-371.
4. Nandi A, Jahagirdar D, Dimitris MC, et al. The impact of parental and medical leave policies on socioeconomic and health outcomes in OECD Countries: A systematic review of the empirical literature. *The Milbank Quarterly.* 2018;96(3):434-471.
5. Campbell M, Katikireddi SV, Hoffmann T, Armstrong R, Waters E, Craig P. TIDieR-PHP: a reporting guideline for population health and policy interventions. *BMJ.* 2018;361.
6. Duvander A-Z, Haas L. Sweden country note. In: Blum S, Koslowski A, Macht A, Moss P, eds. *International Review of Leave Policies and Research 2018* International Network on Leave Policies & Research 2018.
7. *Social insurance in figures 2014.* Försäkringskassan;2014.
8. Duvander A-Z, Mats J. Parental leave use for different fathers: A study of the impact of three Swedish parental leave reforms. In: Eydal GB, Rostgaard T, eds. *Fatherhood in the Nordic Welfare States: Comparing Care Policies and Practice* Policy Press; 2015:347-369.
9. Mussino E, Duvander A-Z, Ma L. Does time count? Immigrant fathers' use of parental leave for a first child in Sweden. *Population.* 2018;73(2):363-382.
10. Försäkringskassan. Parental benefits. 2020; https://www.forsakringskassan.se/privatpers/foralder/nar_barnet_ar_fott/foraldrapenning/!ut/p/z0/04_Sj9CPykssy0xPLMnMz0vMAfIjo8ziTTxcnA3dnQ28_U2DXQwczTwDDcOCXY1CDc31g1Pz9AuyHRUBTbm8uw!!/ Accessed November 11. , 2020
11. Försäkringskassan. Introduction to the Swedish social insurance and Försäkringskassan. 2021; https://www.forsakringskassan.se/english/moving-to-working-studying-or-newly-arrived-in-sweden/introduction-to-the-swedish-social-insurance-and-forsakringskassan. Accessed January 11, 2021.
12. Mussino E, Duvander A-Z. Use It or Save It? Migration background and parental leave uptake in Sweden. *Eur J Popul.* 2016;32(2):189-210.
13. Duvander A-Z. *Family policy in Sweden 2008. Socialförsäkringsrapport.* Försäkringskassan 2008.
14. Andersson G, Hoem JH, Duvander A-Z. Social differentials in speed-premium effects in childbearing in Sweden *Demogr Res.* 2006;14(4):51-70.
15. Duvander A-Z, Johansson M. What are the effects of reforms promoting fathers' parental leave use? *J Eur Soc Policy.* 2012;22(3):319-330.
16. Persson P, Rossin-Slater M. When dad can stay home: fathers' workplace flexibility and maternal health2019. Located at: National Bureau of Economic Research.
17. Duvander A-Z, Ferrini T, Thalberg S. *Swedish parental leave and gender equality. Achievements and reform challenges in a European perspective.* Stockholm, Sweden: Institute for Future Studies;2005.
18. WHO. *Report of a WHO technical consultation on birth spacing.* Geneva: World Health Organization;2005.
19. Davis EM, Babineau DC, Wang X, et al. Short inter-pregnancy intervals, parity, excessive pregnancy weight gain and risk of maternal obesity. *Matern Child Health J.* 2014;18(3):554-562.
20. Johansson K, Wennberg P, Hammarström A. Parental leave and increased physical activity of fathers and mothers—results from the Northern Swedish Cohort. *Eur J Public Health.* 2014;24(6):935-940.

For peer review only - http://bmjopen.bmj.com/site/about/guidelines.xhtml

21. Lee BC, Modrek S, White JS, Batra A, Collin DF, Hamad R. The effect of California's paid family leave policy on parent health: A quasi-experimental study. *Social science & medicine (1982).* 2020;251:112915.
22. Månsdotter A, Lundin A. How do masculinity, paternity leave, and mortality associate? A study of fathers in the Swedish parental & child cohort of 1988/89. *Soc Sci Med.* 2010;71:576–583.
23. Yan J, Liu L, Zhu Y, Huang G, Wang P. The association between breastfeeding and childhood obesity: a meta-analysis. *BMC Public Health.* 2014;14:1267.
24. Wang J, Ramette A, Jurca M, Goutaki M, Beardsmore CS, Kuehni CE. Breastfeeding and respiratory tract infections during the first 2 years of life. *ERJ Open Res.* 2017;3(2):00143-02016.
25. Ekberg J, Eriksson R, Friebel G. Parental leave—A policy evaluation of the Swedish "Daddy-Month" reform. *J Public Econ.* 2013;97:131-143.
26. Karimi A, Lindahl E, Skogman Thoursie P. Labour supply responses to paid parental leave Institute for labor market policy evaluation. In. *FAU Working paper.* Vol 222012.
27. The Helsinki statement on Health in All Policies. *Health Promot Int.* 2014;29(1):17-18.
28. Merton R. The unanticipated consequences of purposive social action. *Am Sociol Rev.* 1936;1:894–904.
29. Portes A. The hidden abode: sociology as analysis of the unexpected: 1999 presidential address. *Am Sociol Rev.* 2000;65(1):1-18.
30. Bonell C, Jamal F, Melendez-Torres GJ, Cummins S. 'Dark logic': theorising the harmful consequences of public health interventions. *J Epidemiol Community Health.* 2015;69(1):95-98.
31. Ludvigsson JF, Otterblad-Olausson P, Pettersson BU, Ekbom A. The Swedish personal identity number: possibilities and pitfalls in healthcare and medical research. *Eur J Epidemiol.* 2009;24:659–667.
32. Ludvigsson JF, Almqvist C, Edstedt Bonamy A-K, et al. Registers of the Swedish total population and their use in medical research. . *Eur J Epidemiol.* 2016;31:125–136.
33. Ekbom A. The Swedish Multi-generation Register. *Methods Mol Biol.* 2011;675:215-220.
34. Ludvigsson JF, Svedberg P, Olén O, Bruze G, Neovius M. The longitudinal integrated database for health insurance and labour market Studies (LISA) and its use in medical research. *Eur J Epidemiol.* 2019;34:423–437.
35. Ludvigsson JF, Andersson E, Ekbom A, et al. External review and validation of the Swedish national inpatient register. *BMC Public Health* 2011;11(450).
36. Cnattingius S, Ericson A, Gunnarskog J, Källen K. A quality study of a medical birth registry. *Scand J Soc Med.* 1990;18:143-148.
37. Brooke HL, Talbäck M, Hörnblad J, et al. The Swedish cause of death register. *Eur J Epidemiol.* 2017;32(9):765-773.
38. Wallerstedt SM, Wettermark B, Hoffmann M. The first decade with the Swedish Prescribed Drug Register - A systematic review of the output in the scientific literature. *Basic Clin Pharmacol Toxicol.* 2016;119(5):464-469.
39. Marsál K, Persson PH, Larsen T, Lilja H, Selbing A, Sultan B. Intrauterine growth curves based on ultrasonically estimated foetal weights. *Acta Paediatr.* 1996;85(7):843-848.
40. Cole TJ, Bellizzi MC, Flegal KM, Dietz WH. Establishing a standard definition for child overweight and obesity worldwide: international survey. *BMJ.* 2000;320:1-6.
41. Cole TJ, Flegal KM, Nicholls D, Jackson AA. Body mass index cut offs to define thinness in children and adolescents: international survey. *BMJ.* 2007;28(335).
42. WHO. Body mass index - BMI. http://www.euro.who.int/en/health-topics/disease-prevention/nutrition/a-healthy-lifestyle/body-mass-index-bmi. Accessed 14-04, 2018.
43. Angrist JD, Pischke JS. *Mastering'metrics: The path from cause to effect.* Princeton University Press; 2014.

For peer review only - http://bmjopen.bmj.com/site/about/guidelines.xhtml

44. Bernal JL, Cummins S, Gasparrini A. Interrupted time series regression for the evaluation of public health interventions: a tutorial. *Int J Epidemiol.* 2017;46(1):348-355.
45. Holowko N, Chaparro M, Nilsson K, et al. Social inequality in pre-pregnancy BMI and gestational weight gain in the first and second pregnancy among women in Sweden. *J Epidemiol Community Health.* 2015;69(12):1154-1161.
46. Hoffman CM, Jeffers S, Carter J, Duthely L, Cotter A, González-Quintero VH. Pregnancy at or beyond age 40 years is associated with an increased risk of fetal and other adverse outcomes. *American Journal of Obstetrics and Gynecology.* 2006:e.11-e.13.
47. Lean SC, Derricott H, Jones RL, Heazell AEP. Advanced maternal age and adverse pregnancy outcomes: A systematic review and meta-analysis. *PLOS ONE.* 2017;12(0):e0186287.
48. Ferguson-Smith MA, Yates JRW. Maternal age specific rates for chromosome aberrations and factors influencing them: Report of a collaborative european study on 52 965 amniocenteses. *Prenatal Diagnosis.* 1984;4(7):5-44.

For peer review only

For peer review only - http://bmjopen.bmj.com/site/about/guidelines.xhtml

Figure legends

Figure 1. Theoretical framework to study the unintended consequences of parental leave policy from a health equity perspective

Figure 2. National registers and policy reforms included in the study

For peer review only

For peer review only - http://bmjopen.bmj.com/site/about/guidelines.xhtml

Figure 1. Theoretical framework to study the unintended consequences of parental leave policy from a health equity perspective

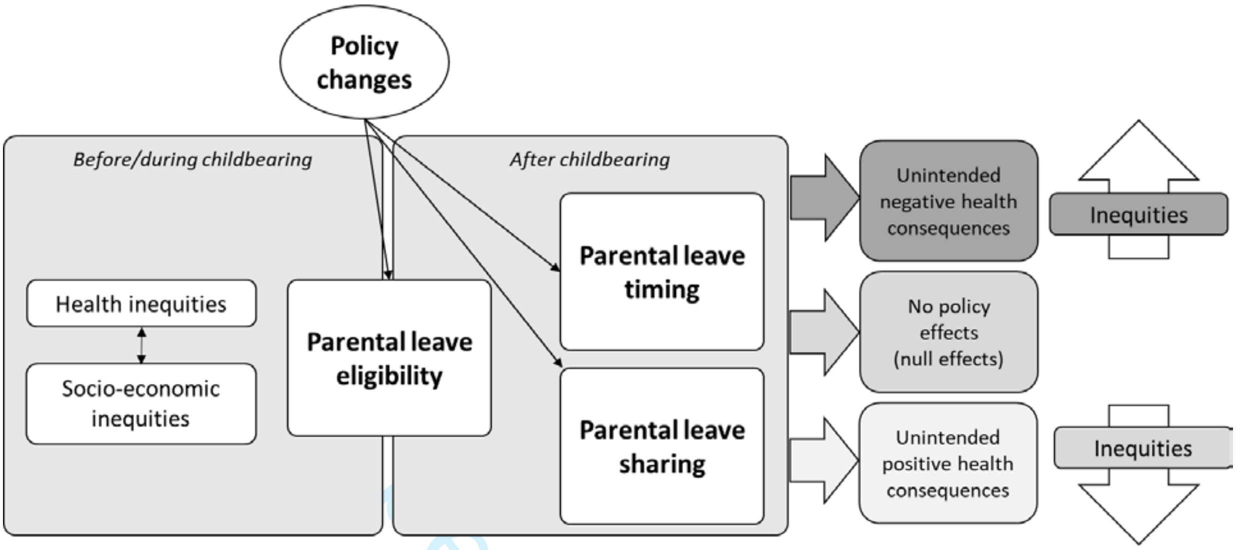

For peer review only

Figure 2. National registers and policy reforms included in the study

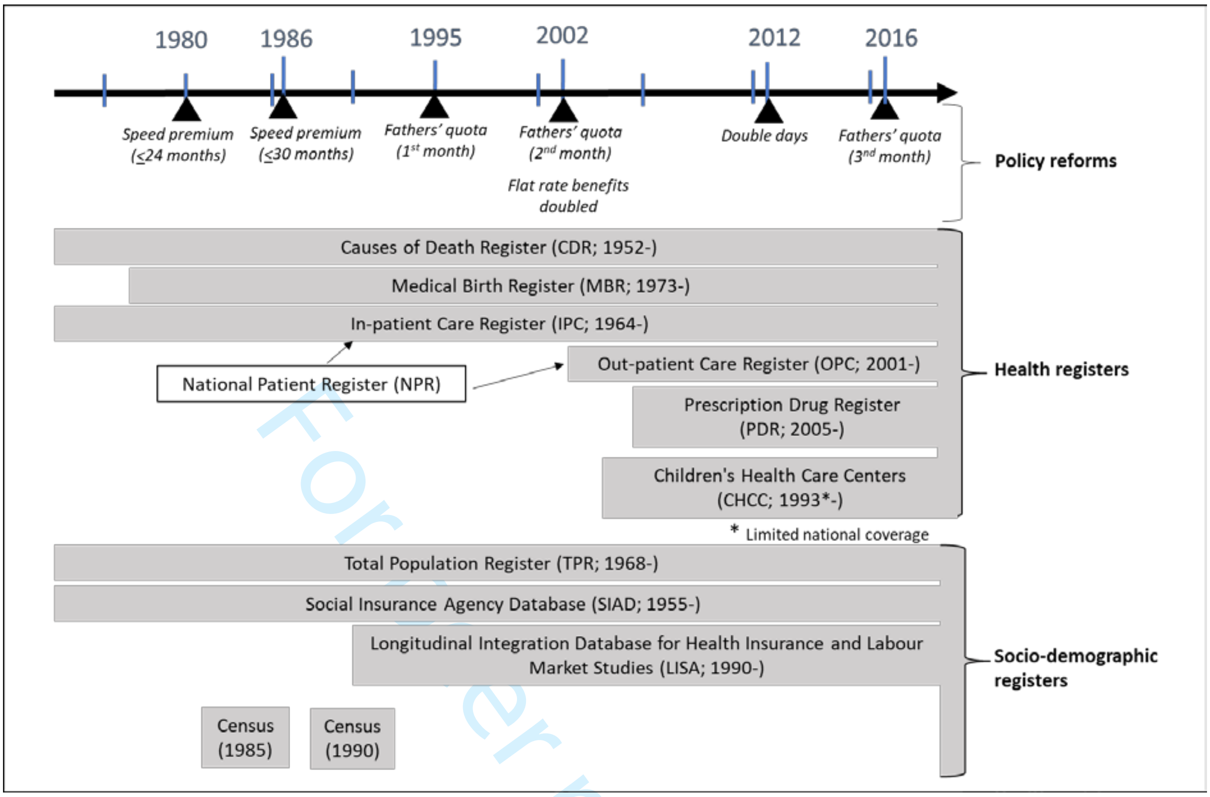

For peer review only

TIDieR-PHP checklist.

Item	Item description	Page in manuscript where item is reported
1 Brief name	Provide the name or a phrase that describes the intervention	**5,6**
2. Why	Describe the logic, mechanisms, or rationale of the intervention, clearly linking intervention elements to the expected effects on immediate or longer term outcomes (or both)	**6-9**
3. What materials	Describe any materials used in the intervention (including online appendices or URLs for further details). For example: — informational materials (may include those provided to recipients of the intervention or in training of intervention providers) — nature and value of any benefit provided (eg, cash, voucher, meal) — any physical resources or infrastructure provided as part of the intervention	**n/a**
4. What and how	Describe how the intervention was planned, established, and intended to be delivered. Depending on the type of intervention, it may be useful to consider: — how sources of funding for the intervention and the service providers were obtained, how users were enrolled and the service delivered — how any payments were made or benefits delivered, how qualifying conditions were implemented — the entity being regulated, the scope of the regulation, permitted level of use; procedures for monitoring or enforcing compliance, and any sanctions for non-compliance — how people were exposed to the intervention, whether it was provided to individuals or larger populations — any underpinning legislation including name, date passed, and legislative body	**4-7**
5. Who provided	Describe the provider of the intervention, including legal status and powers, field organisations and staff responsible for planning, implementation, monitoring and enforcement. Where relevant, describe intervention provider expertise and training (general or specific to the intervention)	**5**
6. Where	Describe the type of location (eg, school, community centre) and the geographical scope of the intervention (eg, national, regional, city-wide). Where relevant, describe the historical, cultural, socioeconomic, or political background to the intervention	**4, 7**
7. When and how often	Describe when the intervention was implemented, how long it remained in place, and, if applicable, the number, duration, and scheduling of occasions	**4-7**
8.1 Planned variation	Describe and provide the reason for any variation or tailoring that was planned or allowed for in the design of the intervention.	**n/a**
8.2 Unplanned variation	Describe and provide the reason for any unplanned variation or modifications in the intervention (eg, between different locations, geographical areas, population subgroups, or over time) that were made after the intervention commenced	**n/a**
9.1 How well	Describe any strategies used or actions taken to maintain fidelity of the intervention (ie, to ensure that the intervention was delivered as intended)	**n/a**
9.2 How well—delivery	Describe the fidelity of the intervention (ie, the extent to which the intervention was delivered as intended)	**6,7**

For peer review only

*If the information is not provided in the primary paper, give details of where this information is available (eg, protocol, other published papers (provide citation details), or a website (provide the URL))

For peer review only

STROBE Statement—checklist of items that should be included in reports of observational studies

	Item No	Recommendation	Page No
**Title and abstract**	1	(*a*) Indicate the study's design with a commonly used term in the title or the abstract	1
		(*b*) Provide in the abstract an informative and balanced summary of what was done and what was found	2
**Introduction**			
Background/rationale	2	Explain the scientific background and rationale for the investigation being reported	1-9
Objectives	3	State specific objectives, including any prespecified hypotheses	10-11, 7-9
**Methods**			
Study design	4	Present key elements of study design early in the paper	1,2, 14-15
Setting	5	Describe the setting, locations, and relevant dates, including periods of recruitment, exposure, follow-up, and data collection	11-15, fig.2,
Participants	6	(*a*) *Cohort study*—Give the eligibility criteria, and the sources and methods of selection of participants. Describe methods of follow-up<br>*Case-control study*—Give the eligibility criteria, and the sources and methods of case ascertainment and control selection. Give the rationale for the choice of cases and controls<br>*Cross-sectional study*—Give the eligibility criteria, and the sources and methods of selection of participants	16
		(*b*) *Cohort study*—For matched studies, give matching criteria and number of exposed and unexposed<br>*Case-control study*—For matched studies, give matching criteria and the number of controls per case	
Variables	7	Clearly define all outcomes, exposures, predictors, potential confounders, and effect modifiers. Give diagnostic criteria, if applicable	12-13
Data sources/ measurement	8*	For each variable of interest, give sources of data and details of methods of assessment (measurement). Describe comparability of assessment methods if there is more than one group	12-13
Bias	9	Describe any efforts to address potential sources of bias	15, 16-17
Study size	10	Explain how the study size was arrived at	n/a
Quantitative variables	11	Explain how quantitative variables were handled in the analyses. If applicable, describe which groupings were chosen and why	n/a
Statistical methods	12	(*a*) Describe all statistical methods, including those used to control for confounding	14, 16-17
		(*b*) Describe any methods used to examine subgroups and interactions	15
		(*c*) Explain how missing data were addressed	16
		(*d*) *Cohort study*—If applicable, explain how loss to follow-up was addressed<br>*Case-control study*—If applicable, explain how matching of cases and controls was addressed	16

For peer review only - http://bmjopen.bmj.com/site/about/guidelines.xhtml

Cross-sectional study—If applicable, describe analytical methods taking account of sampling strategy	
(*e*) Describe any sensitivity analyses	16

Continued on next page

For peer review only

For peer review only - http://bmjopen.bmj.com/site/about/guidelines.xhtml

**Results**			
Participants	13*	(a) Report numbers of individuals at each stage of study—eg numbers potentially eligible, examined for eligibility, confirmed eligible, included in the study, completing follow-up, and analysed	
		(b) Give reasons for non-participation at each stage	
		(c) Consider use of a flow diagram	
Descriptive data	14*	(a) Give characteristics of study participants (eg demographic, clinical, social) and information on exposures and potential confounders	
		(b) Indicate number of participants with missing data for each variable of interest	
		(c) *Cohort study*—Summarise follow-up time (eg, average and total amount)	
Outcome data	15*	*Cohort study*—Report numbers of outcome events or summary measures over time	
		Case-control study—Report numbers in each exposure category, or summary measures of exposure	
		Cross-sectional study—Report numbers of outcome events or summary measures	
Main results	16	(*a*) Give unadjusted estimates and, if applicable, confounder-adjusted estimates and their precision (eg, 95% confidence interval). Make clear which confounders were adjusted for and why they were included	
		(*b*) Report category boundaries when continuous variables were categorized	
		(*c*) If relevant, consider translating estimates of relative risk into absolute risk for a meaningful time period	
Other analyses	17	Report other analyses done—eg analyses of subgroups and interactions, and sensitivity analyses	
**Discussion**			
Key results	18	Summarise key results with reference to study objectives	
Limitations	19	Discuss limitations of the study, taking into account sources of potential bias or imprecision. Discuss both direction and magnitude of any potential bias	
Interpretation	20	Give a cautious overall interpretation of results considering objectives, limitations, multiplicity of analyses, results from similar studies, and other relevant evidence	
Generalisability	21	Discuss the generalisability (external validity) of the study results	
**Other information**			
Funding	22	Give the source of funding and the role of the funders for the present study and, if applicable, for the original study on which the present article is based	

*Give information separately for cases and controls in case-control studies and, if applicable, for exposed and unexposed groups in cohort and cross-sectional studies.

Note: An Explanation and Elaboration article discusses each checklist item and gives methodological background and published examples of transparent reporting. The STROBE checklist is best used in conjunction with this article (freely available on the Web sites of PLoS Medicine at http://www.plosmedicine.org/, Annals of Internal Medicine at http://www.annals.org/, and Epidemiology at http://www.epidem.com/). Information on the STROBE Initiative is available at www.strobe-statement.org.

For peer review only

BMJ Open

Unintended health consequences of Swedish parental leave policy (ParLeHealth): Protocol for a quasi-experimental study

Journal:	*BMJ Open*
Manuscript ID	bmjopen-2021-049682.R1
Article Type:	Protocol
Date Submitted by the Author:	18-May-2021
Complete List of Authors:	Juárez , Sol Pia; Stockholm University, Department of Public Health Sciences; Centre for Health Equity Studies, Stockholm University/Karolinska Institute<br>Honkaniemi, Helena ; Stockholm University, Department of Public Health Sciences<br>Heshmati, Amy; Stockholm University, Department of Public Health Sciences and Centre for Health Equity Studies; Karolinska Institute, Department of Global Public Health<br>Debiasi, Enrico; Stockholm University, Department of Public Health Sciences<br>Dunlavy, Andrea; Stockholm University, Department of Public Health Sciences<br>Hjern, Anders; Stockholm University, Centre for Health Equity Studies; Karolinska Institute, Department of Medicine<br>Rostila, Mikael ; Stockholm University, Department of Public Health Sciences<br>Mussino, Eleonora; Stockholm University, Stockholm University Demography Unit (SUDA)<br>Katikireddi, Srinivasa; University of Glasgow, MRC/CSO Social & Public Health Sciences Unit<br>Duvander, Ann-Zofie; Stockholm University, Stockholm University Demographic Unit (SUDA); Mid University, Department of Sociology
**Primary Subject Heading**:	Public health
Secondary Subject Heading:	Health policy, Sociology
Keywords:	MENTAL HEALTH, Child protection < PAEDIATRICS, PUBLIC HEALTH, REPRODUCTIVE MEDICINE

SCHOLARONE™
Manuscripts

I, the Submitting Author has the right to grant and does grant on behalf of all authors of the Work (as defined in the below author licence), an exclusive licence and/or a non-exclusive licence for contributions from authors who are: i) UK Crown employees; ii) where BMJ has agreed a CC-BY licence shall apply, and/or iii) in accordance with the terms applicable for US Federal Government officers or employees acting as part of their official duties; on a worldwide, perpetual, irrevocable, royalty-free basis to BMJ Publishing Group Ltd ("BMJ") its licensees and where the relevant Journal is co-owned by BMJ to the co-owners of the Journal, to publish the Work in this journal and any other BMJ products and to exploit all rights, as set out in our licence.

The Submitting Author accepts and understands that any supply made under these terms is made by BMJ to the Submitting Author unless you are acting as an employee on behalf of your employer or a postgraduate student of an affiliated institution which is paying any applicable article publishing charge ("APC") for Open Access articles. Where the Submitting Author wishes to make the Work available on an Open Access basis (and intends to pay the relevant APC), the terms of reuse of such Open Access shall be governed by a Creative Commons licence – details of these licences and which Creative Commons licence will apply to this Work are set out in our licence referred to above.

Other than as permitted in any relevant BMJ Author's Self Archiving Policies, I confirm this Work has not been accepted for publication elsewhere, is not being considered for publication elsewhere and does not duplicate material already published. I confirm all authors consent to publication of this Work and authorise the granting of this licence.

For peer review only

Unintended health consequences of Swedish parental leave policy (ParLeHealth): Protocol for a quasi-experimental study

Sol P. Juárez[1,2*] sol.juarez@su.se, ORCID iD: 0000-0001-9086-7588

Helena Honkaniemi[1,2] helena.honkaniemi@su.se, ORCID iD: 0000-0003-0800-0892

Amy Heshmati[1,2,3] amy.heshmati@su.se, ORCID iD: 0000-0002-1462-7328

Enrico Debiasi[1,2] enrico.debiasi@su.se, ORCID iD:0000-0001-5772-5510

Andrea Dunlavy[1,2] andrea.dunlavy@su.se, ORCID iD: 0000-0003-4661-3462

Anders Hjern[1,2,4] anders.hjern@su.se, ORCID iD: 0000-0002-1645-2058

Mikael Rostila[1,2] mikael.rostila@su.se, ORCID iD: 0000-0002-6973-0381

Eleonora Mussino[5] eleonora.mussino@sociology.su.se, ORCID iD: 0000-0002-5311-4277

Srinivasa Vittal Katikireddi[1,2,6] vittal.katikireddi@glasgow.ac.uk, ORCID iD: 0000-0001-6593-9092

Ann-Zofie Duvander[5,7] ann-zofie.duvander@sociology.su.se, ORCID iD: 0000-0001-5241-4588

[1] Department of Public Health Sciences, Stockholm University, Stockholm, Sweden.

[2] Centre for Health Equity Studies, Stockholm University/Karolinska Institutet, Stockholm, Sweden.

[3] Department of Global Public Health, Karolinska Institutet, Stockholm, Sweden.

[4] Department of Medicine, Karolinska Institutet, Stockholm, Sweden

[5] Demographic Unit (SUDA), Department of Sociology, Stockholm University, Stockholm, Sweden.

[6] Institute of Health & Wellbeing. University of Glasgow

[7] Department of Sociology, Mid University, Östersund, Sweden

[*] Corresponding author: Sol P. Juárez, Department of Public Health Sciences, Stockholm University, SE-106 91, Stockholm, Sweden

Word count: 3,994 (up to 4000)
Abstract: 298 (up to 300)
Illustrations: 2
References: 50

For peer review only - http://bmjopen.bmj.com/site/about/guidelines.xhtml

ABSTRACT

Introduction: Sweden has long been praised for a generous parental leave policy oriented towards facilitating a gender-equitable approach to work and parenting. Yet certain aspects of Swedish parental leave could also be responsible for the maintenance of (or even the increase in) health inequalities. Using a 'Health in All Policies' lens, this research project aims to assess the unintended health consequences of various components of Sweden's parental leave policy, including eligibility for and uptake of earnings-based benefits.

Methods and analysis: We will utilise individual-level data from multiple Swedish registers. Sociodemographic information, including on parental leave use, will be retrieved from the Total Population Register, Longitudinal Integration Database for Health Insurance and Labour Market Studies, and Social Insurance Agency Registers. Health information for parents and children will be retrieved from the Patient, Prescribed Drug, Cause of Death, Medical Birth and Children's Health Registers. We will evaluate parents' mental, mothers' reproductive and children's general health outcomes in relation to several policy reforms aiming to protect parental leave benefits in short birth spacing (the *Speed premium*) and to promote father's uptake (the *Father's quota*) and sharing of parental leave days (the *Double days* reform). We will also examine effects of increases in basic parental leave benefit levels. Using quasi-experimental designs, we will compare health outcomes across these reforms and eligibility thresholds with interrupted time series, difference-in-difference, and regression discontinuity approaches to reduce the risk of health selection and assess causality in the link between parental leave use and health.

Ethics and dissemination: This project has been granted all necessary ethical permissions from the Stockholm Regional Ethical Review Board (Dnr 2019-04913) for accessing and analysing de-identified data. The final outputs will primarily be disseminated as scientific articles published in open-access, high-impact peer-reviewed international journals, as well as press releases and policy briefs.

For peer review only

STRENGTHS AND LIMITATIONS OF THIS STUDY

- This will be the first project to evaluate multiple aspects of Swedish parental leave policy by adopting a health equity perspective and Health in All Policies (HiAP) approach.
- The study will utilise quasi-experimental methods on total population register data to assess causal links between parental leave uptake and health.
- The study will expand on existing evidence of health effects by considering outcomes not only for mothers and children, but fathers as well.
- Analyses will be restricted to studying health outcomes that are available within administrative datasets, thereby precluding assessment of potentially more sensitive self-reported assessments of health.

For peer review only

For peer review only - http://bmjopen.bmj.com/site/about/guidelines.xhtml

INTRODUCTION

Childbearing is a life event with significant social and health implications for parents, children and society alike. Among women, childbearing has been considered to be responsible for lower labour market participation and gendered wage gaps (i.e., the 'motherhood penalty'),[1] resulting in lower income protection in old age.[1-3] Childbearing is also a risk factor for various physical and mental health conditions among mothers.[4] By offering secure time off work for childcare and recovery, parental leave has the potential to reduce social and health inequalities in society. However, given that parental leave eligibility and uptake may vary across social strata, it may also have the potential to (re)produce social inequalities in health.

1. *The Swedish parental leave system*

We report intervention details according to the TIDieR-PHP reporting guidelines.[5]

1.1 Length and flexibility of parental leave

Sweden has developed into one of the most generous and flexible paid parental leave systems worldwide.[6] Starting in 1974, parents were entitled to six months of leave to be used within 270 days of birth and shared by the parents as they desired. An additional nine months of leave were introduced incrementally from 1975 to 1989,[6,7] with an extension to use a portion of the leave until the child turned eight years old in 1978. In 1995, one month of the leave was reserved for each parent, a quota which was further extended by one month each in 2002 and 2016.[8,9] Since 2014, couples have been entitled to use 480 parental leave days up until the child turns 12 years old, of which no more than 96 days from the child's fourth birthday.[7]

We will focus on mothers' and fathers' *paid parental leave* (i.e., job-protected leave paid at set benefit levels) rather than *unpaid parental leave* (i.e., job-protected unpaid leave in the first 18 months after birth). Furthermore, we do not examine *maternity leave* (i.e., two weeks of mandatory job-protected leave for the mother prior to or after childbirth, which can be unpaid

For peer review only - http://bmjopen.bmj.com/site/about/guidelines.xhtml

or paid as standard parental leave days), *temporary leave*, including at birth/adoption (formerly *paternity leave*; i.e., up to ten days of separate paid, job-protected leave for partners to attend the delivery or assist the mother with infant care), or leave for the care of sick children.[6,7]

1.2 Parental leave eligibility and compensation

In Sweden, all legally residing parents are eligible to receive parental leave benefits (i.e., job-protected leave), including biological parents as well as individuals with child custody.[10] Individuals must apply for these benefits through the Social Insurance Agency, the governmental authority that decides on and administers insurance and contributions to families and children in Sweden.[11] The benefits are split into general flat-rate or earnings-based benefits, collectively referred to as 'parental benefits' and applied to 390 out of the total 480 days, while the remaining 90 days are paid at a second flat rate, henceforth referred to as 'guarantee days'.[7]

To be eligible for earnings-based parental benefits, an individual must have been employed for at least 240 consecutive days prior to the estimated delivery date with a minimum annual income (82,100 Swedish kronor (SEK) in 2020). Although the remuneration level has varied over time, individuals today receive approximately 80% of their salary up to a certain ceiling (1,006 SEK per day in 2020) from the Social Insurance Agency, with potential top-ups from the employer.[12] If an individual has an income below a certain level (117,590 SEK in 2020) during the 240 days before childbirth, they are only entitled to a general flat-rate benefit (250 SEK per day in 2020) for the first 180 days which they claim for the child. After 180 claimed days, parents can receive benefits based on their current earnings.[7] Finally, if an individual has no income or an annual income below the stipulated level throughout the leave period they will receive the general flat-rate benefit for the full 390 days. However, if an individual is sick, a student, or unemployed but a 'job seeker', they can receive parental leave benefits based on their prior income if they have registered with the Public Employment Service.[10] Regardless of

For peer review only - http://bmjopen.bmj.com/site/about/guidelines.xhtml

employment history, the final 90 days are always paid at a minimum guaranteed level of 60 SEK starting in 1987, and 180 SEK since 2006.[7]

1.2 Policy reforms

The Swedish parental leave system has undergone several reforms over the past half-century. We define a 'policy reform' as any change implemented at a specific time that could impact either uptake or sharing of parental leave, as well as eligibility for earnings-based benefits. To evaluate the unforeseen health consequences of the parental leave policy – i.e., a welfare policy that *does not* primarily target health – we will concentrate on reforms that have either been shown to accomplish their primary aim (i.e., the *Speed premium*, *Father's quota*, and *Double days* reforms) or with undoubtedly significant, albeit unintended, impacts (i.e., the doubled value of general flat-rate benefits).

Doubled flat-rate benefits: Although not explicitly a reform, the doubling of the general flat-rate benefits from 60 SEK per day (from 1987) to 120 SEK per day (in 2002) aimed to secure the economic welfare of low-income families.

Speed premium: Since earnings-related parental leave benefits are estimated based on one's salary prior to childbearing (at approximately 80% of the salary), parents having children in quick succession may choose to temporarily reduce their working hours or be unable to return to work at all, and will thus have lower benefits for their second and subsequent births. In 1980, a supplement (commonly referred to as the "speed premium") was introduced to safeguard women's income between parental leave periods, ensuring that for births with up to 24 months' spacing,[13] and then up to 30 months' spacing (following a 1986 expansion),[14] all earnings-based benefits could be based on the parents' salaries prior to the birth of their previous child, if higher than their current income. An evaluation of the 1986 expansion revealed that an

For peer review only - http://bmjopen.bmj.com/site/about/guidelines.xhtml

increased proportion of Swedish-born parents had children in short succession; even if this was an unintended consequence, the reform has henceforth been called the "speed premium".[14]

Father's quota: Fathers have been eligible to use parental leave since 1974, when parents could choose to divide parental leave days between themselves as they saw fit. However, most days were transferred to and claimed by mothers, with an average use of 320 days in 1994, reinforcing mothers' "double burden" of childcare and work. In response, a *Father's quota* reform was implemented in 1995, reserving a month of leave exclusively for the father, to be forfeited completely if left unused (similarly, a 30-day reserve was allocated to the mother, i.e., a *Mother's quota*). It succeeded in increasing the proportion of fathers using parental leave from 43% to 75%.[15] The *quota* was expanded to two and three months in 2002 and 2016, respectively, but with smaller impacts on uptake.[6]

Double days: In 2012, 30 days of existing parental leave were designated as 'shareable', i.e., could be used simultaneously by both parents within the child's first year of life. By granting flexibility to the couple in their leave use, this "Double days" reform aimed to facilitate the transfer of care from one parent to another. In fact, the reform significantly increased the proportion and number of father's leave days used in the first half-year after childbirth.[16]

All reforms were implemented nationally on January 1st of the indicated year.

2. *Unintended health consequences of the Swedish parental leave system*

Despite the relative generosity of the Swedish parental leave system and its efforts to promote gender equity, some aspects could be responsible for the maintenance or increase in social and health inequities. For example, the strong work-eligibility requirement for earnings-based parental leave benefits may exclude groups with less stability in the labour market, such as students and migrants, from benefits equal to those enjoyed by persons with stable incomes.[17]

Inequities in the work-eligibility requirement can emerge in various ways, either by restricting the parental leave benefits scheme to the securely employed and coincidentally excluding parents with ill health (i.e., *health selection*), or by creating health inequities through the unequal distribution of benefits (i.e., *social causation*). For example, individuals with long-term illness may be more likely to have a weak labour market attachment, which in turn can exclude them from earnings-related parental leave benefits, thus further increasing socioeconomic inequities. Based on this, we expect that the *doubled flat-rate benefits* could increase leave uptake among lower income families or decrease the economic trade-off of using parental leave among lower income families, thus having potential psychosocial benefits.

Parental leave policies might impact health differently following certain policy reforms. For example, the *Speed premium* supplement introduced to secure earnings-based parental leave benefits to parents who have children in quick succession essentially incentivises short birth spacing.[14] This might lead to increased psychosocial stress and adverse reproductive outcomes among women who become pregnant whilst simultaneously rearing an infant or toddler. The WHO recommends a minimum of 24 months, assuming nine months of gestation, before a new conception to avoid adverse reproductive outcomes. [18] Short birth intervals have been associated with nutrition depletion,[19] as the mother might not have enough time between births to recover her nutritional reserves, and higher risks of weight gain and obesity throughout life.[20] In turn, these issues are risk factors for adverse outcomes in subsequent children, including low birthweight, preterm birth and perinatal death.[18,21]

The *Father's quota* was implemented to promote a gender-equitable work-life balance and shared parenting. However, the health consequences of the *Father's quota* have been debated, with a lack of empirical evidence on its differing mechanisms. Studies have shown a positive

For peer review only - http://bmjopen.bmj.com/site/about/guidelines.xhtml

association between the father's parental leave use and healthy behaviours, including decreased alcohol use and increased physical activity,[22,23] as well as decreased mortality risks.[24] Nonetheless, there may be situations in which fathers' leave use is less optimal for or even unfavourable for the family. The quota may have encouraged mothers to return to work sooner, potentially interrupting their physical and psychological recovery from pregnancy and childbirth. These potential adverse effects among parents could extend to the child: shorter maternity leave in the child's first months could shorten breastfeeding, a risk factor for childhood obesity[25] and respiratory track infectious in the first two years of life.[26] However, there is a lack of evidence on changes in mothers' labour supply following the reform,[27,28] suggesting that mothers may have stayed at home with fathers on an unpaid basis, foregoing their income and thus potentially creating psychological strain, especially among low-income parents.

Given that parental leave in Sweden is longer than in most other contexts, it can be argued that any health effect of shortened parental leave among mothers with the introduction of the *Father's quota* would be negligible. However, adverse effects may become apparent in specific sub-populations, including for couples only entitled to flat-rate benefits (that may therefore need to return to work early), as well as individuals with job insecurity or who are self-employed. Identifying adverse health effects in population sub-groups will not only shed light on the differential impacts of parental leave reforms within a population but will have implications for policy transferability in international contexts where parental leave is generally shorter and discussions on reserved days for the father are ongoing.

The adverse unintended consequences of the reserved days for the mother could to some extent be compensated for by the introduction of the subsequent *Double days* reform, which allowed

For peer review only - http://bmjopen.bmj.com/site/about/guidelines.xhtml

parents to use a month of parental leave days *simultaneously* in the crucial first year after birth. In fact, this reform has been shown to have beneficial psychiatric effects for mothers by allowing fathers to participate in childcare as their partners recover from pregnancy and childbirth.[16]

3. Theoretical framework

This project is rooted in the Health in All Policies (HiAP) framework, which advocates for considering the health implications of policies across all sectors.[29] The HiAP approach will help to shed light on the health consequences of a family policy that does not target health, but could have considerable health impacts in society. This approach also highlights how advantages derived from parental leave uptake can be restricted for individuals with poor health, due to the specific work-related requirements for earnings-based benefits. Therefore, in this project health is considered in the sociological perspective of 'unanticipated (or unintended) consequences of social actions',[30] according to which policies and interventions can have latent functional or dysfunctional effects that were not planned or anticipated by relevant actors.[31] This unexpected dimension reveals the complexity of organised social actions, calling for the investigation of well-intentioned interventions to identify non-linear mechanisms through which such consequences arise (see **Figure 1**). Within this framework, the persistence of (or increase in) health inequalities associated with parental leave policy can be seen as an adverse side effect to be identified and addressed through policy reform.[32]

4. Aim/objectives and research questions

For peer review only - http://bmjopen.bmj.com/site/about/guidelines.xhtml

The aim of this project is to evaluate the consequences of parental leave policy in Sweden from a health equity perspective. More specifically, the project will cover the following research questions (RQ):

RQ#1. Is eligibility for earnings-based parental leave benefits associated with parents' mental health?

RQ#2. Is short birth spacing in relation to the *Speed premium* supplement reform associated with health problems in the subsequent pregnancy of the mother (early pregnancy obesity) and the subsequent child (low birthweight, preterm and small-for-gestational age)?

RQ#3. Does the introduction and expansion of the *Father's quota* reserve days influence the mental health of parents after the birth of their child?

RQ#4. Does the introduction of simultaneous parental leave use through the *Double days* reform influence parents' mental health?

RQ#5: Does the introduction of the *Father's quota* and *Double days* reforms influence children's health (episodes of respiratory tract infections, childhood obesity) or health-related outcomes (duration of breastfeeding)?

METHODS AND ANALYSIS

Study population

We will include all Swedish- and foreign-born individuals registered in Sweden between 1973 and 2019 and their Swedish-born children, followed through their first five years of life. For most studies, we will focus on a subsample consisting of all women (and their partners) residing in Sweden who had their first birth in the country between 1973 and 2019.

For peer review only - http://bmjopen.bmj.com/site/about/guidelines.xhtml

Studies will be primarily focused on primiparous couples, with secondary analyses for multiparous couples. We will only include biological parents, since we aim to examine joint biological and psychosocial consequences of parental leave uptake, with singleton children, given that parents of multiple births are entitled to more days of leave.[6] Separate analyses by parents' sociodemographic characteristics, nativity, and cohabitation status will be conducted to investigate the heterogeneity of effects in the population.

Data

This project will use data from available national registers, collected and linked by the Swedish authorities. Using a unique personal identity number,[33] we will identify and reconstruct families in the study population through the Total Population Register (TPR; 1968-)[34] and the Multigenerational Register (MGR; 2001-).[35] Sociodemographic information will be retrieved from the TPR, the Longitudinal Integration Database for Health Insurance and Labour Market Studies (LISA; 1990-),[36] the Swedish Social Insurance Agency Database (SIAD; 1955-) and the 1985 and 1990 Population and Housing Censuses. We will link health information from the National Patient Register (NPR), on inpatient (IPR; 1964-)[37] and outpatient care (IPR; 2001-); and the Medical Birth (MBR; 1973-),[38] Cause of Death (1952-)[39] and Prescribed Drug (2005-) Registers.[40] We also plan to include information from the Swedish Children's Health Centres (CHC; 1993-), with full coverage of longitudinal childhood obesity and breastfeeding data from the counties of Uppsala and Örebro (see **Figure 2**).

Outcomes

Child health outcomes include low birthweight (<2,500 grams, irrespective of gestational age), preterm birth (<37 gestational weeks), and a small-for-gestational age measure (<10th percentile of birthweight for each gestational age, based on sex-specific Swedish reference

For peer review only - http://bmjopen.bmj.com/site/about/guidelines.xhtml

curve for normal foetal growth)[41] from the MBR; hospitalisation episodes of respiratory tract infections during the first two years of life (International Classification of Diseases-10 [ICD-10] Codes J20.5, J21.0, J21.9, J20.9, J18.9, J12.9 and J22.9) from the NPR; and duration of partial or exclusive breastfeeding (at months 2, 4, 6, 9 and 12) as well as childhood obesity (body mass index [BMI; weight in kilograms/height in meters^2] using age- and sex-specific cut-offs for children's overweight and obesity[42,43] at 18 months and 3, 4 and 5 years old) from the CHR. Maternal health outcomes include maternal early pregnancy overweight (BMI 25-29.9 kg/m^2) and obesity (BMI 30-39.9 kg/m^2).[44]

Mental health outcomes will be assessed for both parents using count data on purchases of prescription anxiolytic and antidepressant medications (Anatomical Therapeutic Classification or ATC Codes N05B and N06A, respectively) as well as outpatient visits and hospitalisations with a primary diagnosis of mental and behavioural disorders (until 1997, ICD-9 Codes 290-319; thereafter, ICD-10 Codes F00-F99), from childbirth to 18 and 36 months after birth.

Outcomes will be expressed as prevalence or rates, given availability of follow-up time.

Covariates

Annual information on parental leave uptake (number of days) for each parent will also be available from LISA. Specific information on parental leave eligibility and uptake (including length of paid and unpaid leave) will be retrieved from the SIAD.

Demographic characteristics will include parents' age at childbirth, country of birth (i.e., Swedish vs. other and specific origins), and marital and cohabitation status, from the TPR and LISA. Sociodemographic characteristics including educational attainment, household

For peer review only - http://bmjopen.bmj.com/site/about/guidelines.xhtml

disposable income, individual labour income, employment status and receipt of sick leave benefits will be drawn from LISA.

Study design and statistical analyses

A variety of advanced designs and statistical methods for observational data will be applied. First, using descriptive and correlational designs, we will implement standard multivariable methods to quantify links between pre-birth health conditions, parental leave eligibility and uptake, and post-birth health outcomes while accounting for confounding using relevant covariates and heterogeneity analyses.

Second, we will use robust quasi-experimental approaches to assess the causal effects of parental leave policy on health, reducing the risk of selection effects and unmeasured confounding by harnessing the natural, "as-if" randomisation around policies.[45,46] With this approach, we can isolate the absolute policy effects on health even in the absence of detailed information on changes in parental leave uptake. These approaches include interrupted time series (ITS), regression discontinuity (RD) and difference-in-difference (DiD) designs. For ITS designs, segmented regression models will be adjusted for seasonality and trend while accounting for autocorrelation.[46] For RD designs, continuity around the cut-off will be tested using the McCrary density test and similarity of eligible and ineligible observations will be determined through descriptive statistics. Finally, for DiD designs, we will examine the data to confirm parallel trends and other relevant assumptions.[45]

RQ#1: We will examine the effects of the 2002 *doubling of flat-rate benefits* on the mental health of parents. An RD design will be applied to plot psychiatric hospitalisation rates with labour income as a forcing variable, with separate plots before (with an income cut-off equivalent to 75 SEK, at 80% reimbursement rate) and after 2002 (with a cut-off equivalent to

For peer review only - http://bmjopen.bmj.com/site/about/guidelines.xhtml

150 SEK daily). Cut-offs for both parents' income will be examined. We will triangulate our findings with a DiD approach, comparing those eligible for the increased benefits to those ineligible before and after reforms.

RQ#2: A segmented-regression ITS of monthly birth data comparing the rate of low birthweight and preterm births three years before and after the 1980 and 1986 *Speed premium* reforms will be implemented. We will also compare the prevalence of obesity in mothers early in their second pregnancy before and after the reform.

RQ#3: We will conduct an ITS analysis of parents of children born before and after the 1995 *Father's quota* reform using segmented regression to capture changes in psychiatric hospitalisation rates up to 36 months after childbirth, based on fathers' eligibility for the *Father's quota*.

RQ#4: An ITS for parents to children born before and after the 2012 *Double days* reform will be implemented to capture changes in the number of outpatient visits for mental health and psychotropic prescriptions within 36 months after childbirth, by child's month of birth.

RQ#5: We will implement separate ITS analyses of children born before and after the 1995 *Father's quota* and 2012 *Double days* reforms, comparing hospitalisation rates for diagnoses of respiratory tract infections (in children under 2), prevalence of childhood obesity (at 18 months and 3, 4 and 5 years old), and changes in breastfeeding duration.

Additional analyses

Given the potential for effect modification and compositional changes in the study populations, sub-group analyses by parents' sociodemographic characteristics (i.e., annual labour income, to determine eligibility for earnings-based benefits, or educational attainment) will be considered for all research questions. Analyses by parents' nativity will be implemented for RQ#2-4, given differential responses to the policies by Swedish- and foreign-born parents.[14]

For peer review only - http://bmjopen.bmj.com/site/about/guidelines.xhtml

Other relevant parental characteristics, i.e., age at childbirth and cohabitation status, will also be considered.

Sensitivity analyses will be performed to account for systematically missing data, such as maternal weight (i.e., given that women appearing to be of normal weight are less monitored for obesity).[47] For studies examining first births, sensitivity analyses restricting mental health outcomes to the 18 months post-birth will be considered in order to exclude the potential health effects related to a second pregnancy.

For all ITS designs, we will specify multiple intervention points to assess the possibility of lagged policy effects. Robustness analyses of falsification ('pseudo-intervention') dates, and falsification outcomes (e.g., pre-birth hospitalisations) will also be considered.

Limitations

Despite our reliance on a robust quasi-experimental approach, it is difficult to predict the relative timing of the behavioural response to each parental leave reform (i.e., in uptake) and its health consequences. Parents have been shown to respond quickly to policy changes,[14,15,27] but given that some parents may not be aware of their eligibility on the reform dates, delayed reaction times could lead to lagged health effects for sub-groups such as migrants.[12] Thus, we will utilise a long follow-up both within the life course (i.e., years since birth) and in the context of the policy (i.e., years since reform implemented).

Quasi-experimental designs have the potential to reduce unmeasured confounding, but policies themselves, as well as ongoing trends, could inadvertently modify the composition of the study populations, potentially biasing any causal interpretations. For one, the *Speed premium* may have affected the composition of individuals who decided to have children after the reform, i.e., encouraging more parents eligible for earnings-based benefits to have children after 1986, given their increased level of benefits for subsequent children. Exogenous impacts other than

For peer review only - http://bmjopen.bmj.com/site/about/guidelines.xhtml

the policy under investigation could also contribute to changing population compositions, as in the case of the 1990s economic crisis, which may have discouraged couples with lower socioeconomic position (SEP) from having children after the 1995 *Father's quota*. We will thus examine varying sociodemographic characteristics of the study populations before and after each reform to assess the robustness of the design to approximate causal effects, with corresponding sub-group and sensitivity analyses.

Registers generally have greater coverage than other data sources but may be prone to bias in the form of missing data and over-coverage (i.e., unreported outmigration), which we will address in appropriate sensitivity analyses. The historical availability of register data may also influence our analytical choices. For example, to evaluate the impact of the *Father's quota* on psychiatric health, we will primarily look at the first reserved month introduced in 1995.[6] However, only severe health outcomes (psychiatric hospitalisations) can be assessed with the available registers at that time. In order to evaluate less severe outcomes (outpatient visit and prescription data) of increased fathers' leave use, we will evaluate the more recent *Double days* reform.[16]

Finally, although our primary aim is to evaluate unintended adverse health effects of these parental leave reforms, we will also consider health benefits. For example, short birth intervals promoted by the *Speed premium* supplement could also have led to women having subsequent children at younger age, thereby reducing the risk for adverse reproductive outcomes associated with advanced age (e.g., foetal abnormalities, preterm and stillbirths).[48-50]

By considering both negative and positive health outcomes, in conjunction with a variety of parental leave reforms and parental characteristics, this study will openly explore future directions for international parental leave research.

For peer review only - http://bmjopen.bmj.com/site/about/guidelines.xhtml

Patient and public involvement

This project will not involve any patients or the public. However, we will attempt to communicate the findings of the study to the public through the mass media.

Ethics and dissemination

Although we handle personal information, individual consent is not required since the information is anonymised by the agencies responsible for data protection, and none of the records will have personal identifiers attached. Statistics Sweden may also aggregate some information (such as country of birth) to ensure that individuals cannot be identified in relation to other variables (e.g., education or place of residence). Nevertheless, since our dataset contains individual-level information and thus requires special provisions for its use, we applied for all necessary ethical permissions from the Stockholm Regional Ethical Review Board to cover the specific research questions addressed by this project (Dnr 2019-04913).

Author's contribution

SPJ, HH and AD conceived and designed the study with feedback from EM and AZD (regarding the policy), MR and AH (regarding health outcomes and data access) and HH and SVK (regarding methodology). SPJ drafted the manuscript with help from HH, ED and AMH and all authors made substantial contributions. All authors approved the final version submitted for publication. SPJ is the guarantor.

Funding statement

This work was supported by the Swedish Research Council (VR) grant number 2018-01825 and the Swedish Council for Health, Working Life, and Social welfare (FORTE) Grant number 2016–07128. In addition, SVK acknowledges funding from a NRS Senior Clinical fellowship (SCAF/15/02), the Medical Research Council (MC_UU_12017/13) and the Scottish Government Chief Scientist Office (SPHSU13).

Funders of the study had no involvement in study design and will not be involved in any parts of the study, including data collection, data analysis, data interpretation, or writing of the report.

Competing Interests Statement: None

For peer review only

REFERENCES

1. Budig MJ, England P. The wage penalty for motherhood. *Am Sociol Rev.* 2001;66(2):204-225.
2. England P. Gender inequality in labor markets: The role of motherhood and segregation. *Social Politics: International Studies in Gender, State & Society.* 2005;12(2):264-288.
3. Charles M. A world of difference: international trends in women's economic status. *Annu Rev Sociol.* 2011;37:355-371.
4. Nandi A, Jahagirdar D, Dimitris MC, et al. The impact of parental and medical leave policies on socioeconomic and health outcomes in OECD Countries: A systematic review of the empirical literature. *The Milbank Quarterly.* 2018;96(3):434-471.
5. Campbell M, Katikireddi SV, Hoffmann T, Armstrong R, Waters E, Craig P. TIDieR-PHP: a reporting guideline for population health and policy interventions. *BMJ.* 2018;361.
6. Duvander A-Z, Haas L. Sweden country note. In: Blum S, Koslowski A, Macht A, Moss P, eds. *International Review of Leave Policies and Research 2018* International Network on Leave Policies & Research 2018.
7. *Social insurance in figures 2014.* Försäkringskassan;2014.
8. Duvander A-Z, Mats J. Parental leave use for different fathers: A study of the impact of three Swedish parental leave reforms. In: Eydal GB, Rostgaard T, eds. *Fatherhood in the Nordic Welfare States: Comparing Care Policies and Practice* Policy Press; 2015:347-369.
9. Mussino E, Duvander A-Z, Ma L. Does time count? Immigrant fathers' use of parental leave for a first child in Sweden. *Population.* 2018;73(2):363-382.
10. Försäkringskassan. Parental benefits. 2020; https://www.forsakringskassan.se/privatpers/foralder/nar_barnet_ar_fott/foraldrapenning/!ut/p/z0/04_Sj9CPykssy0xPLMnMz0vMAfIjo8ziTTxcnA3dnQ28_U2DXQwczTwDDcOCXY1CDc31g1Pz9AuyHRUBTbm8uw!!/ Accessed November 11. , 2020
11. Försäkringskassan. Introduction to the Swedish social insurance and Försäkringskassan. 2021; https://www.forsakringskassan.se/english/moving-to-working-studying-or-newly-arrived-in-sweden/introduction-to-the-swedish-social-insurance-and-forsakringskassan. Accessed January 11, 2021.
12. Mussino E, Duvander A-Z. Use It or Save It? Migration background and parental leave uptake in Sweden. *Eur J Popul.* 2016;32(2):189-210.
13. Duvander A-Z. *Family policy in Sweden 2008. Socialförsäkringsrapport.* Försäkringskassan 2008.
14. Andersson G, Hoem JH, Duvander A-Z. Social differentials in speed-premium effects in childbearing in Sweden *Demogr Res.* 2006;14(4):51-70.
15. Duvander A-Z, Johansson M. What are the effects of reforms promoting fathers' parental leave use? *J Eur Soc Policy.* 2012;22(3):319-330.
16. Persson P, Rossin-Slater M. When dad can stay home: fathers' workplace flexibility and maternal health2019. Located at: National Bureau of Economic Research.
17. Duvander A-Z, Ferrini T, Thalberg S. *Swedish parental leave and gender equality. Achievements and reform challenges in a European perspective.* Stockholm, Sweden: Institute for Future Studies;2005.
18. WHO. *Report of a WHO technical consultation on birth spacing.* Geneva: World Health Organization;2005.
19. King JC. The risk of maternal nutritional depletion and poor outcomes increases in early or closely spaced pregnancies. *J Nutr.* 2003;133(5):1732s-1736s.
20. Davis EM, Babineau DC, Wang X, et al. Short inter-pregnancy intervals, parity, excessive pregnancy weight gain and risk of maternal obesity. *Matern Child Health J.* 2014;18(3):554-562.

For peer review only - http://bmjopen.bmj.com/site/about/guidelines.xhtml

21. Conde-Agudelo A, Rosas-Bermúdez A, Kafury-Goeta A. Birth spacing and risk of adverse perinatal outcomes: a meta analysis. *JAMA.* 2006;295(15):1809-1823.
22. Johansson K, Wennberg P, Hammarström A. Parental leave and increased physical activity of fathers and mothers—results from the Northern Swedish Cohort. *Eur J Public Health.* 2014;24(6):935-940.
23. Lee BC, Modrek S, White JS, Batra A, Collin DF, Hamad R. The effect of California's paid family leave policy on parent health: A quasi-experimental study. *Social science & medicine (1982).* 2020;251:112915.
24. Månsdotter A, Lundin A. How do masculinity, paternity leave, and mortality associate? A study of fathers in the Swedish parental & child cohort of 1988/89. *Soc Sci Med.* 2010;71:576–583.
25. Yan J, Liu L, Zhu Y, Huang G, Wang P. The association between breastfeeding and childhood obesity: a meta-analysis. *BMC Public Health.* 2014;14:1267.
26. Wang J, Ramette A, Jurca M, Goutaki M, Beardsmore CS, Kuehni CE. Breastfeeding and respiratory tract infections during the first 2 years of life. *ERJ Open Res.* 2017;3(2):00143-02016.
27. Ekberg J, Eriksson R, Friebel G. Parental leave—A policy evaluation of the Swedish "Daddy-Month" reform. *J Public Econ.* 2013;97:131-143.
28. Karimi A, Lindahl E, Skogman Thoursie P. Labour supply responses to paid parental leave Institute for labor market policy evaluation. *FAU Working paper.* Vol 222012.
29. The Helsinki statement on Health in All Policies. *Health Promot Int.* 2014;29(1):17-18.
30. Merton R. The unanticipated consequences of purposive social action. *Am Sociol Rev.* 1936;1:894–904.
31. Portes A. The hidden abode: sociology as analysis of the unexpected: 1999 presidential address. *Am Sociol Rev.* 2000;65(1):1-18.
32. Bonell C, Jamal F, Melendez-Torres GJ, Cummins S. 'Dark logic': theorising the harmful consequences of public health interventions. *J Epidemiol Community Health.* 2015;69(1):95-98.
33. Ludvigsson JF, Otterblad-Olausson P, Pettersson BU, Ekbom A. The Swedish personal identity number: possibilities and pitfalls in healthcare and medical research. *Eur J Epidemiol.* 2009;24:659–667.
34. Ludvigsson JF, Almqvist C, Edstedt Bonamy A-K, et al. Registers of the Swedish total population and their use in medical research. . *Eur J Epidemiol.* 2016;31:125–136.
35. Ekbom A. The Swedish Multi-generation Register. *Methods Mol Biol.* 2011;675:215-220.
36. Ludvigsson JF, Svedberg P, Olén O, Bruze G, Neovius M. The longitudinal integrated database for health insurance and labour market Studies (LISA) and its use in medical research. *Eur J Epidemiol.* 2019;34:423–437.
37. Ludvigsson JF, Andersson E, Ekbom A, et al. External review and validation of the Swedish national inpatient register. *BMC Public Health* 2011;11(450).
38. Cnattingius S, Ericson A, Gunnarskog J, Källen K. A quality study of a medical birth registry. *Scand J Soc Med.* 1990;18:143-148.
39. Brooke HL, Talbäck M, Hörnblad J, et al. The Swedish cause of death register. *Eur J Epidemiol.* 2017;32(9):765-773.
40. Wallerstedt SM, Wettermark B, Hoffmann M. The first decade with the Swedish Prescribed Drug Register - A systematic review of the output in the scientific literature. *Basic Clin Pharmacol Toxicol.* 2016;119(5):464-469.
41. Marsál K, Persson PH, Larsen T, Lilja H, Selbing A, Sultan B. Intrauterine growth curves based on ultrasonically estimated foetal weights. *Acta Paediatr.* 1996;85(7):843-848.
42. Cole TJ, Bellizzi MC, Flegal KM, Dietz WH. Establishing a standard definition for child overweight and obesity worldwide: international survey. *BMJ.* 2000;320:1-6.

For peer review only - http://bmjopen.bmj.com/site/about/guidelines.xhtml

For peer review only

43. Cole TJ, Flegal KM, Nicholls D, Jackson AA. Body mass index cut offs to define thinness in children and adolescents: international survey. *BMJ.* 2007;28(335).
44. WHO. Body mass index - BMI. http://www.euro.who.int/en/health-topics/disease-prevention/nutrition/a-healthy-lifestyle/body-mass-index-bmi. Accessed 14-04, 2018.
45. Angrist JD, Pischke JS. *Mastering'metrics: The path from cause to effect.* Princeton University Press; 2014.
46. Bernal JL, Cummins S, Gasparrini A. Interrupted time series regression for the evaluation of public health interventions: a tutorial. *Int J Epidemiol.* 2017;46(1):348-355.
47. Holowko N, Chaparro M, Nilsson K, et al. Social inequality in pre-pregnancy BMI and gestational weight gain in the first and second pregnancy among women in Sweden. *J Epidemiol Community Health.* 2015;69(12):1154-1161.
48. Hoffman CM, Jeffers S, Carter J, Duthely L, Cotter A, González-Quintero VH. Pregnancy at or beyond age 40 years is associated with an increased risk of fetal and other adverse outcomes. *American Journal of Obstetrics and Gynecology.* 2006:e.11-e.13.
49. Lean SC, Derricott H, Jones RL, Heazell AEP. Advanced maternal age and adverse pregnancy outcomes: A systematic review and meta-analysis. *PLOS ONE.* 2017;12(0):e0186287.
50. Ferguson-Smith MA, Yates JRW. Maternal age specific rates for chromosome aberrations and factors influencing them: Report of a collaborative european study on 52 965 amniocenteses. *Prenatal Diagnosis.* 1984;4(7):5-44.

For peer review only

Figure legends

Figure 1. Theoretical framework to study the unintended consequences of parental leave policy from a health equity perspective

Figure 2. National registers and policy reforms included in the study

For peer review only

For peer review only - http://bmjopen.bmj.com/site/about/guidelines.xhtml

Figure 1. Theoretical framework to study the unintended consequences of parental leave policy from a health equity perspective

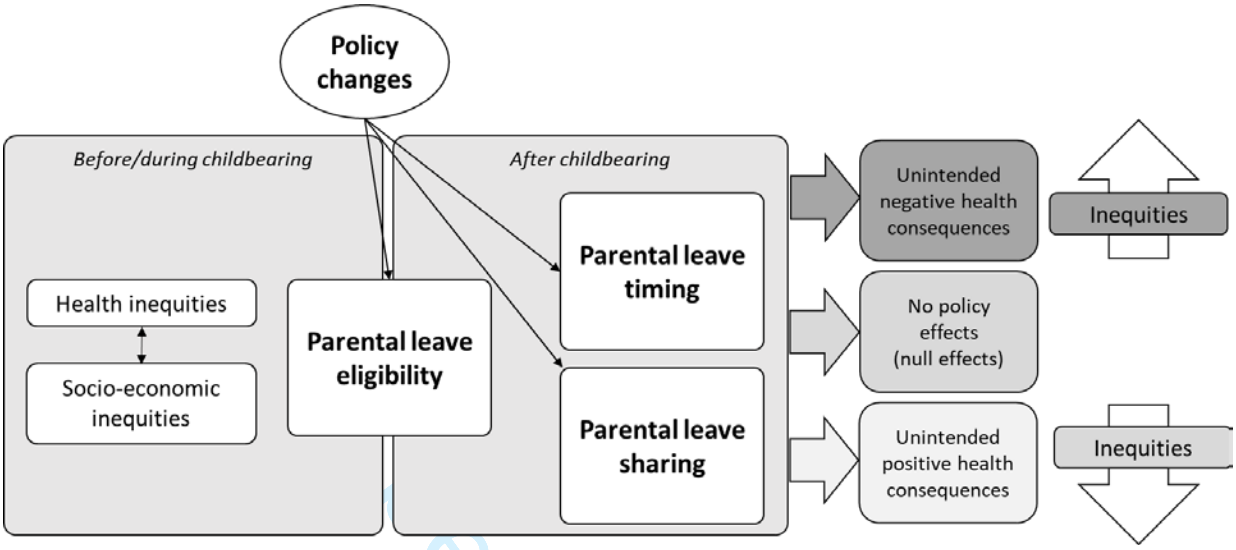

For peer review only

Figure 2. National registers and policy reforms included in the study

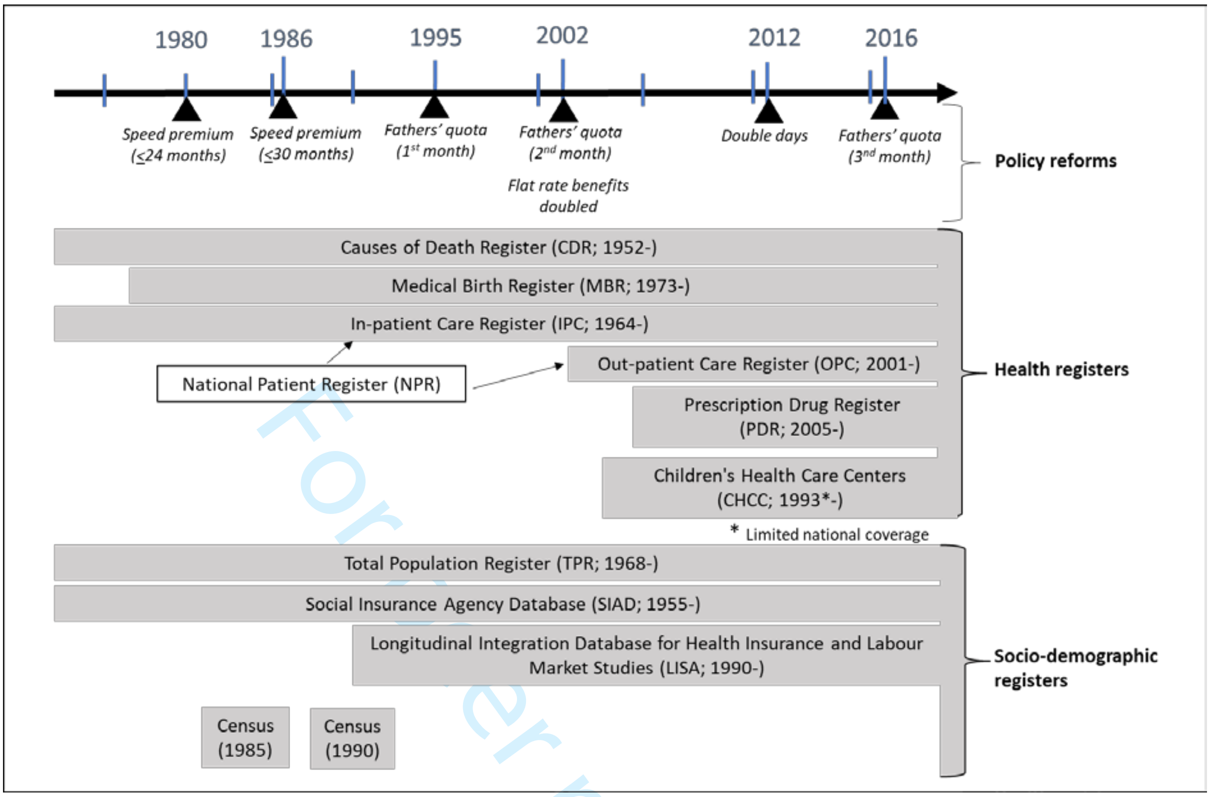

For peer review only

TIDieR-PHP checklist.

Item	Item description	Page in manuscript where item is reported
1 Brief name	Provide the name or a phrase that describes the intervention	**5,6**
2. Why	Describe the logic, mechanisms, or rationale of the intervention, clearly linking intervention elements to the expected effects on immediate or longer term outcomes (or both)	**6-9**
3. What materials	Describe any materials used in the intervention (including online appendices or URLs for further details). For example: — informational materials (may include those provided to recipients of the intervention or in training of intervention providers) — nature and value of any benefit provided (eg, cash, voucher, meal) — any physical resources or infrastructure provided as part of the intervention	**n/a**
4. What and how	Describe how the intervention was planned, established, and intended to be delivered. Depending on the type of intervention, it may be useful to consider: — how sources of funding for the intervention and the service providers were obtained, how users were enrolled and the service delivered — how any payments were made or benefits delivered, how qualifying conditions were implemented — the entity being regulated, the scope of the regulation, permitted level of use; procedures for monitoring or enforcing compliance, and any sanctions for non-compliance — how people were exposed to the intervention, whether it was provided to individuals or larger populations — any underpinning legislation including name, date passed, and legislative body	**4-7**
5. Who provided	Describe the provider of the intervention, including legal status and powers, field organisations and staff responsible for planning, implementation, monitoring and enforcement. Where relevant, describe intervention provider expertise and training (general or specific to the intervention)	**5**
6. Where	Describe the type of location (eg, school, community centre) and the geographical scope of the intervention (eg, national, regional, city-wide). Where relevant, describe the historical, cultural, socioeconomic, or political background to the intervention	**4, 7**
7. When and how often	Describe when the intervention was implemented, how long it remained in place, and, if applicable, the number, duration, and scheduling of occasions	**4-7**
8.1 Planned variation	Describe and provide the reason for any variation or tailoring that was planned or allowed for in the design of the intervention.	**n/a**
8.2 Unplanned variation	Describe and provide the reason for any unplanned variation or modifications in the intervention (eg, between different locations, geographical areas, population subgroups, or over time) that were made after the intervention commenced	**n/a**
9.1 How well	Describe any strategies used or actions taken to maintain fidelity of the intervention (ie, to ensure that the intervention was delivered as intended)	**n/a**
9.2 How well—delivery	Describe the fidelity of the intervention (ie, the extent to which the intervention was delivered as intended)	**6,7**

For peer review only

*If the information is not provided in the primary paper, give details of where this information is available (eg, protocol, other published papers (provide citation details), or a website (provide the URL))

For peer review only

STROBE Statement—checklist of items that should be included in reports of observational studies

	Item No	Recommendation	Page No
**Title and abstract**	1	(*a*) Indicate the study's design with a commonly used term in the title or the abstract	1
		(*b*) Provide in the abstract an informative and balanced summary of what was done and what was found	2
**Introduction**			
Background/rationale	2	Explain the scientific background and rationale for the investigation being reported	1-9
Objectives	3	State specific objectives, including any prespecified hypotheses	10-11, 7-9
**Methods**			
Study design	4	Present key elements of study design early in the paper	1,2, 14-15
Setting	5	Describe the setting, locations, and relevant dates, including periods of recruitment, exposure, follow-up, and data collection	11-15, fig.2,
Participants	6	(*a*) *Cohort study*—Give the eligibility criteria, and the sources and methods of selection of participants. Describe methods of follow-up<br>*Case-control study*—Give the eligibility criteria, and the sources and methods of case ascertainment and control selection. Give the rationale for the choice of cases and controls<br>*Cross-sectional study*—Give the eligibility criteria, and the sources and methods of selection of participants	16
		(*b*) *Cohort study*—For matched studies, give matching criteria and number of exposed and unexposed<br>*Case-control study*—For matched studies, give matching criteria and the number of controls per case	
Variables	7	Clearly define all outcomes, exposures, predictors, potential confounders, and effect modifiers. Give diagnostic criteria, if applicable	12-13
Data sources/ measurement	8*	For each variable of interest, give sources of data and details of methods of assessment (measurement). Describe comparability of assessment methods if there is more than one group	12-13
Bias	9	Describe any efforts to address potential sources of bias	15, 16-17
Study size	10	Explain how the study size was arrived at	n/a
Quantitative variables	11	Explain how quantitative variables were handled in the analyses. If applicable, describe which groupings were chosen and why	n/a
Statistical methods	12	(*a*) Describe all statistical methods, including those used to control for confounding	14, 16-17
		(*b*) Describe any methods used to examine subgroups and interactions	15
		(*c*) Explain how missing data were addressed	16
		(*d*) *Cohort study*—If applicable, explain how loss to follow-up was addressed<br>*Case-control study*—If applicable, explain how matching of cases and controls was addressed	16

For peer review only

Cross-sectional study—If applicable, describe analytical methods taking account of sampling strategy	
(e) Describe any sensitivity analyses	16

Continued on next page

For peer review only

For peer review only - http://bmjopen.bmj.com/site/about/guidelines.xhtml

**Results**			
Participants	13*	(a) Report numbers of individuals at each stage of study—eg numbers potentially eligible, examined for eligibility, confirmed eligible, included in the study, completing follow-up, and analysed	
		(b) Give reasons for non-participation at each stage	
		(c) Consider use of a flow diagram	
Descriptive data	14*	(a) Give characteristics of study participants (eg demographic, clinical, social) and information on exposures and potential confounders	
		(b) Indicate number of participants with missing data for each variable of interest	
		(c) *Cohort study*—Summarise follow-up time (eg, average and total amount)	
Outcome data	15*	*Cohort study*—Report numbers of outcome events or summary measures over time	
		Case-control study—Report numbers in each exposure category, or summary measures of exposure	
		Cross-sectional study—Report numbers of outcome events or summary measures	
Main results	16	(*a*) Give unadjusted estimates and, if applicable, confounder-adjusted estimates and their precision (eg, 95% confidence interval). Make clear which confounders were adjusted for and why they were included	
		(*b*) Report category boundaries when continuous variables were categorized	
		(*c*) If relevant, consider translating estimates of relative risk into absolute risk for a meaningful time period	
Other analyses	17	Report other analyses done—eg analyses of subgroups and interactions, and sensitivity analyses	
**Discussion**			
Key results	18	Summarise key results with reference to study objectives	
Limitations	19	Discuss limitations of the study, taking into account sources of potential bias or imprecision. Discuss both direction and magnitude of any potential bias	
Interpretation	20	Give a cautious overall interpretation of results considering objectives, limitations, multiplicity of analyses, results from similar studies, and other relevant evidence	
Generalisability	21	Discuss the generalisability (external validity) of the study results	
**Other information**			
Funding	22	Give the source of funding and the role of the funders for the present study and, if applicable, for the original study on which the present article is based	

*Give information separately for cases and controls in case-control studies and, if applicable, for exposed and unexposed groups in cohort and cross-sectional studies.

Note: An Explanation and Elaboration article discusses each checklist item and gives methodological background and published examples of transparent reporting. The STROBE checklist is best used in conjunction with this article (freely available on the Web sites of PLoS Medicine at http://www.plosmedicine.org/, Annals of Internal Medicine at http://www.annals.org/, and Epidemiology at http://www.epidem.com/). Information on the STROBE Initiative is available at www.strobe-statement.org.

For peer review only
