# Supplementary material for: Unintended health consequences of Swedish parental leave policy (ParLeHealth): protocol for a quasi-experimental study
Source: BMJ Open. 2021 Jun 9;11(6):e049682. doi: 10.1136/bmjopen-2021-049682 (PMC8191630; doi:10.1136/bmjopen-2021-049682)
Supplement: Reviewer comments [file bmjopen-2021-049682.reviewer_comments.pdf]

PEER REVIEW HISTORY

BMJ Open publishes all reviews undertaken for accepted manuscripts. Reviewers are asked to complete a checklist review form (**http://bmjopen.bmj.com/site/about/resources/checklist.pdf**) and are provided with free text boxes to elaborate on their assessment. These free text comments are reproduced below.

ARTICLE DETAILS

**TITLE (PROVISIONAL)**	Unintended health consequences of Swedish parental leave policy (ParLeHealth): Protocol for a quasi-experimental study
**AUTHORS**	Juárez , Sol Pia; Honkaniemi, Helena; Heshmati, Amy; Debiasi, Enrico; Dunlavy, Andrea; Hjern, Anders; Rostila, Mikael; Mussino, Eleonora; Katikireddi, Srinivasa; Duvander, Ann-Zofie

VERSION 1 – REVIEW

**REVIEWER**	Bezruchka, Stephen<br>University of Washington Seattle Campus, Global Health & Health Services
**REVIEW RETURNED**	23-Mar-2021

**GENERAL COMMENTS**	Your study is set up to add to the literature on the value of various forms of paid parental leave that is mostly lacking in today's scholarship.

**REVIEWER**	Chuang, Hung-Yi<br>Kaohsiung Medical University Hospital, Department of Occupational and Environmental Medicine
**REVIEW RETURNED**	30-Mar-2021

**GENERAL COMMENTS**	I have no more comment for the authors. Thanks for giving me the chance to review the project.
